# Spontaneous mutation in *2310061I04Rik* results in reduced expression of mitochondrial genes and impaired brain myelination

**DOI:** 10.1371/journal.pone.0290487

**Published:** 2024-12-04

**Authors:** Erdyni N. Tsitsikov, Khanh P. Phan, Yufeng Liu, Alla V. Tsytsykova, Rosalia Paterno, David M. Sherry, Anthony C. Johnson, Ian F. Dunn

**Affiliations:** 1 Department of Neurosurgery, University of Oklahoma Health Sciences Center, Oklahoma City, OK, United States of America; 2 Neuroscience Ph.D. Program, University of Oklahoma Health Sciences Center, Oklahoma City, OK, United States of America; 3 Department of Cell Biology, University of Oklahoma Health Sciences Center, Oklahoma City, OK, United States of America; 4 Department of Pharmaceutical Sciences, University of Oklahoma Health Sciences Center, Oklahoma City, OK, United States of America; 5 Department of Physiology, University of Oklahoma Health Sciences Center, Oklahoma City, OK, United States of America; Indian Institute of Technology Indore, INDIA

## Abstract

Here, we describe a spontaneous mouse mutant with a deletion in a predicted gene *2310061I04Rik* (*Rik*) of unknown function located on chromosome 17. A 59 base pair long deletion occurred in the first intron of the *Rik* gene and disrupted its expression. *Rik*^*null*^ mice were born healthy and appeared anatomically normal up to two weeks of age. After that, these mice showed inhibited growth, ataxic gait, and died shortly after postnatal day 24 (P24). Transcriptome analysis at P14 and P23 revealed significantly reduced expression of mitochondrial genes in *Rik*^*null*^ brains compared to wild type controls including *mt-Nd4*, *mt-Cytb*, *mt-Nd2*, *mt-Co1*, *mt-Atp6*, and others. Similarly, genes specific for myelinating oligodendrocytes also showed reduced expression in P23 *Rik*^*null*^ brains compared to controls. Histological examination of anterior thalamic nuclei demonstrated decreased myelination of anteroventral nuclei but not of anterodorsal nuclei in P23 *Rik*^*null*^ mice. Myelination of the anterior commissure was also impaired and displayed extensive vacuolation. Consistently with these findings, immunohistochemistry showed reduced expression of Opalin, a glycoprotein expressed in differentiated oligodendrocytes. Taken together, these results suggest that RIK is important for oligodendrocyte maturation and myelination in the developing brain.

## Introduction

Myelin is a specialized wrapping of tightly compacted layers of specialized plasma membrane that surrounds axons in the central and peripheral nervous system and is derived from oligodendrocytes in the central nervous system and Schwann cells in the peripheral nervous system [1,2]. Myelin serves as electrical insulation that prevents decay of action potentials as they travel between the unmyelinated nodes of Ranvier along the axon. Myelin also provides trophic support to the axon. Disruption of myelin structure or energetics impairs axonal signal transmission and is a key component of several devastating neurodegenerative diseases, including multiple sclerosis, amyotrophic lateral sclerosis, progressive multifocal leukoencephalopathy, central pontine and extrapontine myelinosis, and others [3]. The biological mechanisms that regulate the formation, maintenance, and function of myelin remain incompletely understood. Identification of genes and proteins important to the formation, maintenance, and energetics of myelin is important to understanding the basic biology of myelin formation and function, demyelinating disease pathology, and development of new therapeutic interventions for these devastating diseases.

Neuroscience has been greatly advanced by the availability of mutant mouse models. For many years, various spontaneous mutations and polymorphic alleles provided the only source for genetic studies. Classic neurological mouse mutants include *shiverer* [4], *quaking* [5], *reeler* [6], *pcd* [7] and many others, which have been critical tools for understanding the function of specific genes in the central nervous system. Affected homozygotes of *shiverer* (*shi*) mice develop characteristic "shaking" or "shivering" gait approximately two weeks after birth. This shivering increases in severity with age, and mice die prematurely, typically between 50 and 100 days after birth [8]. The central nervous system (CNS) of the mutant mouse is hypomyelinated but the peripheral nervous system (PNS) appears normal. These mice fail to make myelin due to a deletion of the gene, encoding myelin basic protein (MBP) [8].

Homozygous *quaking viable* (*qk*^*v*^) mice display vigorous tremors starting at about postnatal day 10 (P10), especially pronounced in hind limbs, and experience convulsive tonic-clonic seizures as they mature [5,9]. These mice have pronounced demyelination in both CNS and PNS, resulting from reduced numbers of myelin lamellae and failure of the resulting myelin to compact properly [10]. The original *qk*^*v*^ allele resulted from a large spontaneous deletion that affects three loci on chromosome 17 (chr17): *parkin RBR E3 ubiquitin protein ligase* (*Prkn*), *Park2* co-regulated (*Pacrg*), and *quaking*, *KH domain containing RNA binding* (*Qki*), which is highly expressed in oligodendrocytes (OLs) and astrocytes in CNS as well as Schwann cells in PNS [11]. Further analysis revealed that the alterations in *Qki* locus are responsible for neurological phenotype of *qk*^*v*^ mice [12]. Forward genetics experiments generated additional *Qki* locus mutants with varying phenotypes depending on genetic environment of any given allele. Those phenotypes are also compounded by complex multigenic differences among commonly used mouse strains and substrains [13,14].

In this study, we describe a serendipitous mutation in a predicted gene, *2310061I04Rik*, which causes a neurological phenotype similar to those associated with myelination defects caused by mutations. *Rik* has no previously described function and is positioned on chr17 just ~25MB downstream from the *Qki* locus. A 59-bp deletion in the first intron of the *Rik* gene spontaneously arose in one of the embryonic cell (ES) clones during generation of floxed *TNF receptor-associated factor 7* (*Traf7*^*fl/fl*^*)* mice and resulted in *Rik* expression deficiency. *Rik*^*null*^ mice were born healthy, but by two weeks of age were smaller than their littermates, exhibited ataxic gait, and died before 4 weeks of age. While P14 *Rik*^*null*^ mice demonstrated normal expression of genes specific for differentiated oligodendrocyte, they had lower expression of mitochondrial genes in compared to wild type controls. At P23, *Rik*^*null*^ mice displayed decreased expression of mitochondrial genes as well as myelinating oligodendrocyte genes. Histological and immunohistochemical studies revealed reduced myelination in *Rik*^*null*^ brains.

## Results

### Spontaneous deletion in *2310061I04Rik* gene

To investigate the physiological function of TRAF7 *in vivo*, we generated mice with a conditionally targeted *Traf7* allele [15]. The conditional allele contained loxP sites flanking exons 2 and 14 in the *Traf7* gene. Heterozygous *Traf7*-floxed allele (*Traf7*^*+/fl*^) mice were healthy and fertile. Homozygous *Traf7*^*fl/fl*^ pups appeared normal at birth, but unexpectedly stopped gaining weight after postnatal day 12 (S1A and S1B Fig) and developed a pronounced ataxia with seizure-like behavior (Movie 1) and had to be euthanized shortly after P30 (S1C Fig). For clarity, *Traf7*-floxed mice showing pathology are referred to as *Traf7*^fl*/fl*^ mice to distinguish them from healthy *Traf7*^*fl/fl*^ animals, which were described previously in Tsitsikov et al. [15]. To determine whether *Traf7*^fl*/fl*^ mice had floxed allele-linked spontaneous mutation(s) in protein coding regions, we performed whole exome sequencing using tail DNA from two P30 *Traf7*^fl*/fl*^ animals that showed pathology and two C57BL/6 mice of the same age. None of *Traf7*^fl*/fl*^ animals revealed relevant homozygous sequence variations in the coding region of genes located in the vicinity of *Traf7* on chr17.

**Fig 1:**
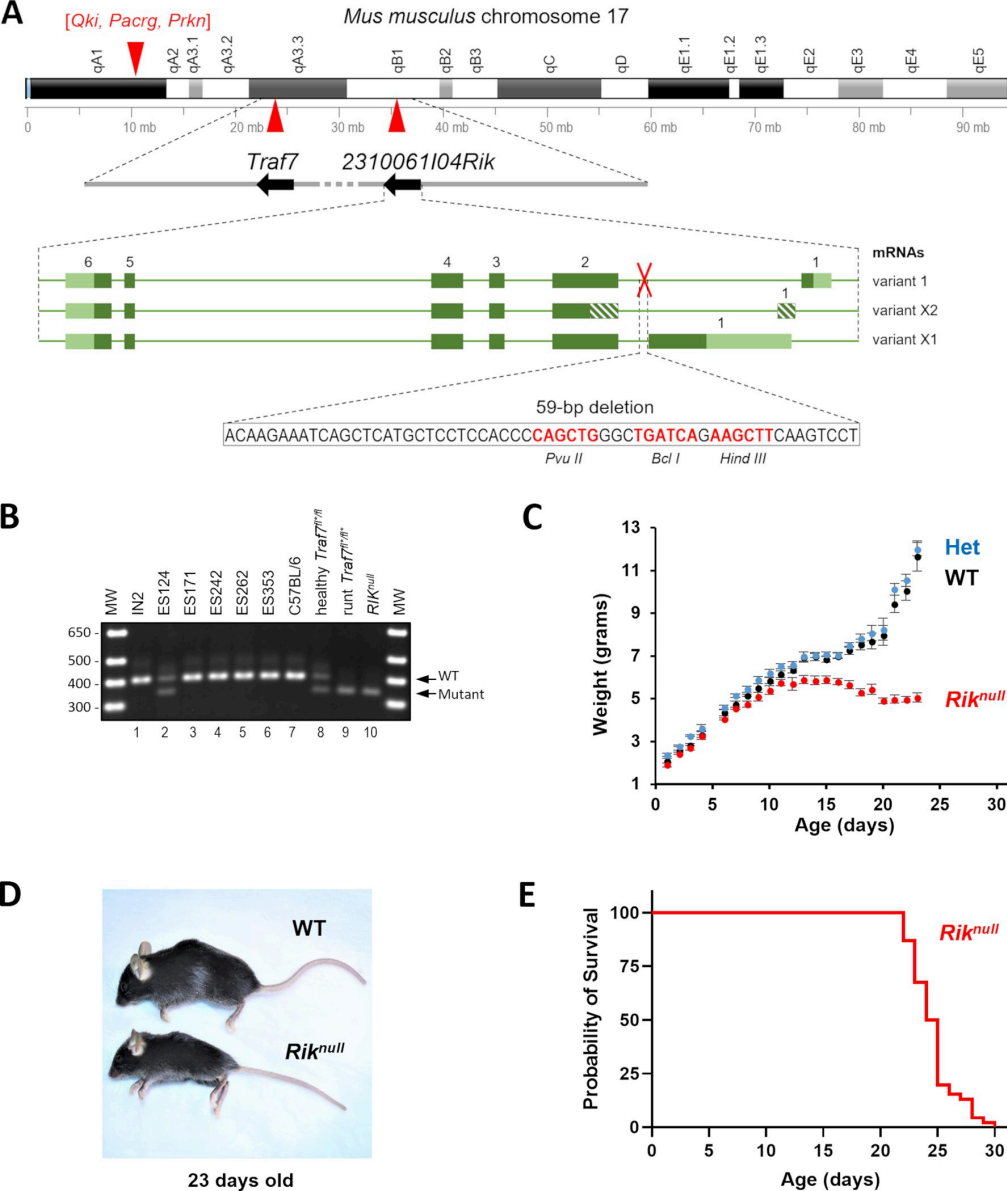
Severe runting and early death of mice with serendipitous deletion in *2310061I04Rik* gene. (**A**) Schematic drawing (up-to-scale) of the mouse chromosome 17. Location of *2310061I04Rik* and *Traf7* genes on the chromosome are marked by red triangles and their directions of transcription are shown by black arrows on grey line in a blowout drawing of their loci below. Predicted variants of mRNA transcribed from the *2310061I04Rik* gene are depicted on the bottom panel in green color. Exons are presented as green boxes with coding (dark green) and non-coding (striped or light green) regions. Location of the deletion is marked by a red cross and its sequence is shown below the mRNA scheme in a blow-out window. Restriction sites within the deletion region are shown in red font with corresponding enzymes in *Italic* font underneath. (**B**) Detection of WT (401 bp) and mutant *Rik* (342 bp) alleles in ES cell line (IN2), ES clones with *Traf7*-floxed alleles, and *Traf7*^fl*/fl*^ and *Rik*^*null*^ mice by conventional PCR and agarose gel electrophoresis. MW: Molecular Weight markers (1 Kb Plus Ladder). (**C**) Mouse weight chart from birth to end of life of *Rik*^*null*^ mice (n = 175 WT (2–20 animals), n = 348 HET (4–45 animals), n = 145 (2–21 animals) *Rik*^*null*^, median±SEM). (**D**) Representative 23 days old WT and *Rik*^*null*^ mice. (**E**) Probability of survival of *Rik*^*null*^ mice (n = 45).

Next, we produced mice with whole body deletion of *Traf7* by crossing *Traf7-floxed* mice with *E2a-Cre*-transgenic animals [16]. While *Traf7*^-*/+^ mice were phenotypically normal and fertile, *Traf7*^-*/-*^ animals died around day 10 of embryonic development (E10) [15]. To understand gene expression differences between WT and *Traf7*^-*/-*^ embryos, we compared their transcriptome profiles at E9.5 by RNA sequencing (RNA-seq) (Supplemental file 1). The analysis revealed that the WT embryos formed a tight cluster following principal component analysis (PCA) (S2A Fig), but the distribution of the *Traf7*^-*/-*^ embryos did not cluster tightly, with three samples near the WT cluster, suggesting a recent branching of transcriptional profiles. Indeed, most identified genes (11,755) were common between these two groups (S2B Fig). Only 277 and 163 of the differentially expressed genes (DEGs) were specifically expressed in either *Traf7*^-*/-*^ or WT embryos, respectively. Hierarchical clustering based on gene expression profiles (Heat map) revealed high similarity of expression patterns between samples within each group and substantial differences between them (S2C Fig). As expected, *Traf7* showed the greatest significant difference in relative expression between WT embryos and their *Traf7*^-*/-*^ counterparts (S2D Fig). The DEG with the largest difference in relative expression and high statistical significance was *Kruppel-like transcription factor 2* (*Klf2*), a key blood flow-responsive gene in endothelial cells [17]. Interestingly, one of the DEGs was the predicted *2310061I04Rik* gene (S2D Fig), which has an unknown function and is located on chr17 approximately 11MB downstream of *Traf7* gene (Fig 1A). Detailed analysis with the Integrative Genomics Viewer (IGV) demonstrated a 59-base pair (bp) deletion in the first intron of the *2310061I04Rik* gene, 125 bp upstream of exon 2. Curiously, this region contains recognition sequences for three commonly used restriction endonucleases Hind III, Pvu II, and Bcl I (Fig 1A). To examine the origin of this 59-bp deletion, we designed a genotyping PCR assay around this deletion and tested embryonic stem (ES) cell clones, which were used for blastocyst injection during generation of the *Traf7*^*fl/+*^ mice. The ES124 clone was heterozygous for the *2310061I04Rik* mutation, but four other clones (ES171, ES242, ES262, and ES353) and the paternal ES cell line IN2 used for transfection, possessed no such deletion (Fig 1B, lanes 1 to 7). The *2310061I04Rik* gene was first sequenced and predicted by the RIKEN (Designated National Research and Development Institute in Japan) mouse genome encyclopedia project [18]. Because *2310061I04Rik* has no designated name or described function, for convenience we temporarily named this gene “*Rik”*, even though it is a common suffix in names of many unknown genes identified by RIKEN.

### A 59-bp deletion in *2310061I04Rik* gene confers the runt phenotype

To explore whether the runt phenotype of *Traf7*^fl*/fl*^ pups arose due to impaired expression of *Rik*, we bred *Traf7*^+/fl*^ animals with C57BL/6 mice for several generations to unlink the mutated *Rik* and *LoxP3*-modified *Traf7* loci. The resulting mutant mice had a *Rik* deletion but an intact *Traf7* gene (*Rik*^*null*^). As shown in Fig 1B, healthy *Traf7*^fl/fl*^ mice carried only one allele with *Rik* deletion (lane 8), while both *Traf7*^fl*/fl*^ and *Rik*^*null*^ mice were homozygous for the *Rik*^*null*^ allele and displayed a similar runt phenotype (lanes 9–10). Mice heterozygous for *Rik* deletion were phenotypically indistinguishable from their WT littermates, indicating that the *Rik*^*null*^ allele was a recessive carrier of the runt phenotype. Homozygous *Rik*^*null*^ mice were born at expected Mendelian inheritance distribution ratio. They displayed no obvious abnormalities at birth and, like the original runt *Traf7*^fl*/fl*^ mice, *Rik*^*null*^ pups gained weight similar to WT littermates until P12 (Fig 1C), when usually the eyes are open, fur growth is complete, and teeth are erupted. After this time point, WT pups progressively gained weight and grew in size by eating more solid food (JAX Mice Pup Appearance by Age (nih.gov)). *Rik*^*null*^ mice had the normal numbers of teeth and eyes, but started to lose weight after P12, exhibited ataxic gait, and died around P24, one week earlier than runt *Traf7*^fl*/fl*^ mice (Fig 1D and 1E, and Movie 2). In contrast, *Traf7*^*fl/fl*^ mice without *Rik* deletion were healthy and fertile as described in Tsitsikov et al. [15].

### P14 *Rik*^*null*^ brains have an abnormal transcriptional profile

Because the *Rik*^*null*^ mouse phenotype was consistent with CNS abnormalities, we compared brain transcriptome profiles from 14 days old littermate WT and *Rik*^*null*^ pups to gain insight into the developmental differences at the earliest time point of the weight gain bifurcation. The PCA plot of the RNA-seq analysis displayed that both control and *Rik*^*null*^ samples did not form compact clusters (S3A Fig). However, there was a good separation between them, with only one of the eight *Rik*^*null*^ samples located near WT controls. The vast majority of identified genes (13,623) were common between the groups, with only 317 and 230 DEGs upregulated in WT or *Rik*^*null*^ brains, respectively (S3B Fig). The Heat map revealed high sample similarity within each group and a good separation between the groups (S3C Fig).

**Fig 2:**
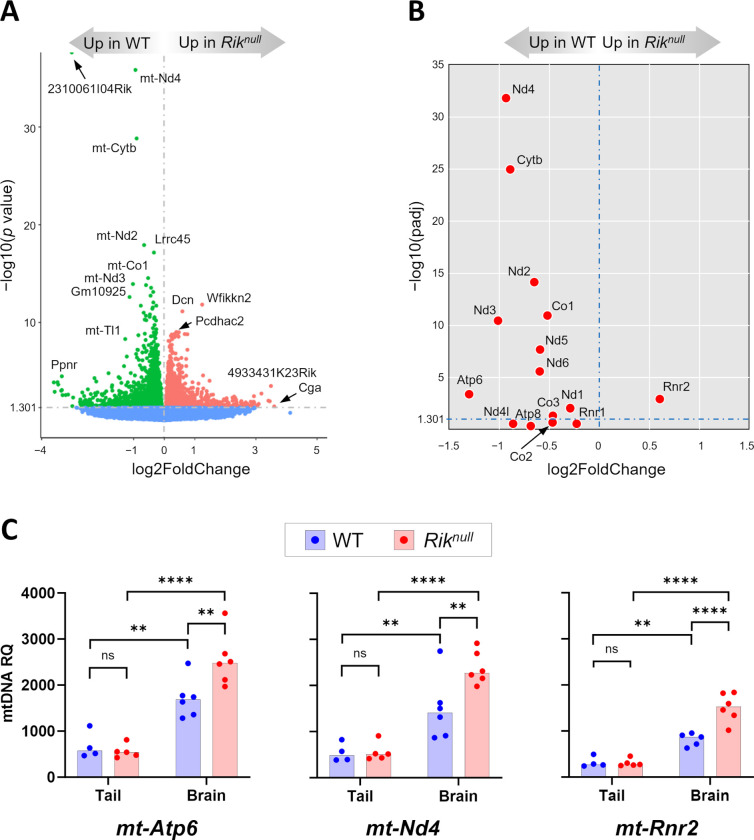
Analysis of 14 days old *Rik*^*null*^ brain transcriptome. (**A**) Volcano plot of RNA-seq analysis visualizing significant DEGs in P14 *Rik*^*null*^ versus WT brains. Magnitude of change (x-axis) vs. statistically significant *p* values (y-axis). Points that have a *p* value less than 0.05 (−log10 = 1.301) are shown in blue (n = 6 WT, n = 5 *Rik*^*null*^). (**B**) Scatter plot of RNA expression levels from 13 messenger and 2 ribosomal RNA genes located in mitochondrial DNA: *Rik*^*null*^ versus WT relative expression (x-axis) vs. statistical significance (y-axis) of the difference. (**C**) Relative quantity (RQ) of mtDNA in brains and tails in WT and *Rik*^*null*^ mice. RQ of three representative mitochondrial genes were measured by gene-specific quantitative real time PCR using nuclear DNA-coded *Gapdh* gene as internal control. Two-factor (genotype, tissue) ANOVA with Bonferroni post-hoc test. n = 4–6 WT, n = 5–6 *Rik*^*null*^. ** p < 0.01, *** p < 0.001, **** p < 0.0001, ns—non-significant.

Remarkably, the expression of *Rik* in *Rik*^*null*^ brains decreased more than 8-fold and showed the greatest difference in relative expression compared to WT counterparts (Fig 2A). In contrast, the expression of closely positioned neighboring genes *alpha tubulin acetyltransferase 1* (*Atat1*) and *DEAH-box helicase 16* (*Dhx16*) was not inhibited (Supplemental file 2), indicating that the 59-bp deletion only interfered with *Rik* expression and, therefore, might contain gene-specific regulatory elements important for its transcription.

Five of the top seven DEGs with the highest significant difference between *Rik*^*null*^ and control mice were genes encoded by mitochondrial DNA, including *mt-Nd4*, *mt-Cytb*, *mt-Nd2*, *mt-Co1*, and *mt-Nd3* (Fig 2A). Because these genes encode subunits of three mitochondrial proton pumps, we examined the expression of all 13 messenger and two ribosomal RNAs (Fig 2B). Of these 15 genes, 14 showed reduced expression in *Rik*^*null*^ brains, with only *mt-Rnr2*, which encodes a large subunit of ribosomal 16S RNA, showing increased expression. Thus, the *Rik*^*null*^ mutation caused a substantial reduction in the expression of mitochondrial genes. While the highest statistical significance was observed for *mt-Nd4*, a subunit of mitochondrial respiratory chain complex I, the biggest decline in expression was displayed by *mt-Atp6*, encoding a subunit of the F_1_F_0_ATP-synthase complex (Fig 2B). Accordingly, gene set enrichment analysis using the Kyoto Encyclopedia of Genes and Genomes (KEGG) pathway database unveiled higher expression of thermogenesis and reactive oxygen species genes in WT controls compared to mutants (S4A Fig). In contrast, genes associated with the phosphatidylinositol/protein kinase B (PI3-Akt) signaling pathway, human papilloma virus infection, and axon guidance were significantly enriched in *Rik*^*null*^ brains (S4B Fig). The PI3-Akt pathway and axon guidance proteins are both important for proper CNS axon growth [19]. Enriched expression of these genes in the brains of *Rik*^*null*^ mice into the third week of postnatal development, when axonal growth should be mostly completed in altricial rodents such as mice and rats [20], is consistent with white matter anomalies noted in *Rik*^*null*^ mice (see below).

To examine whether the difference in brain expression of mitochondrial genes between *Rik*^*null*^ and WT mice was due to different mitochondrial DNA (mtDNA) copy number per cell (CN), we examined mtDNA CN by measuring the relative quantity (RQ) of three representative mitochondrial genes by gene-specific quantitative real time PCR (qPCR). As internal control we used *Gapdh*, which is coded by nuclear DNA and present in two copies in each mononucleated cell. We chose *mt-Atp6*, *mt-Nd4*, and *mt-Rrn2*, because the first two genes demonstrated decreased mRNA expression in *Rik*^*null*^ brains compared to WT counterparts, while the third one displayed the opposite result, an increased expression in *Rik*^*null*^ brains compared to controls (Fig 2B). We also compared the RQ of mtDNA between tails and brains in the same groups of mice. The experiments revealed that mtDNA of each of these genes was no different between *Rik*^*null*^ and WT tails (Fig 2C). In contrast, *Rik*^*null*^ brains demonstrated significantly higher mtDNA content compared to WT brains for all three genes, independent of mRNA expression levels of each of these genes. Thus, these results suggested that P14 *Rik*^*null*^ brains have higher mtDNA copy numbers compared to controls. Interestingly, there was significantly higher RQ of mtDNA in the brains compared to tails in *Rik*^*null*^ mice as well as in WT animals.

### Low expression of genes specific for myelinating oligodendrocytes in P23 *Rik*^*null*^ brains

**Fig 3:**
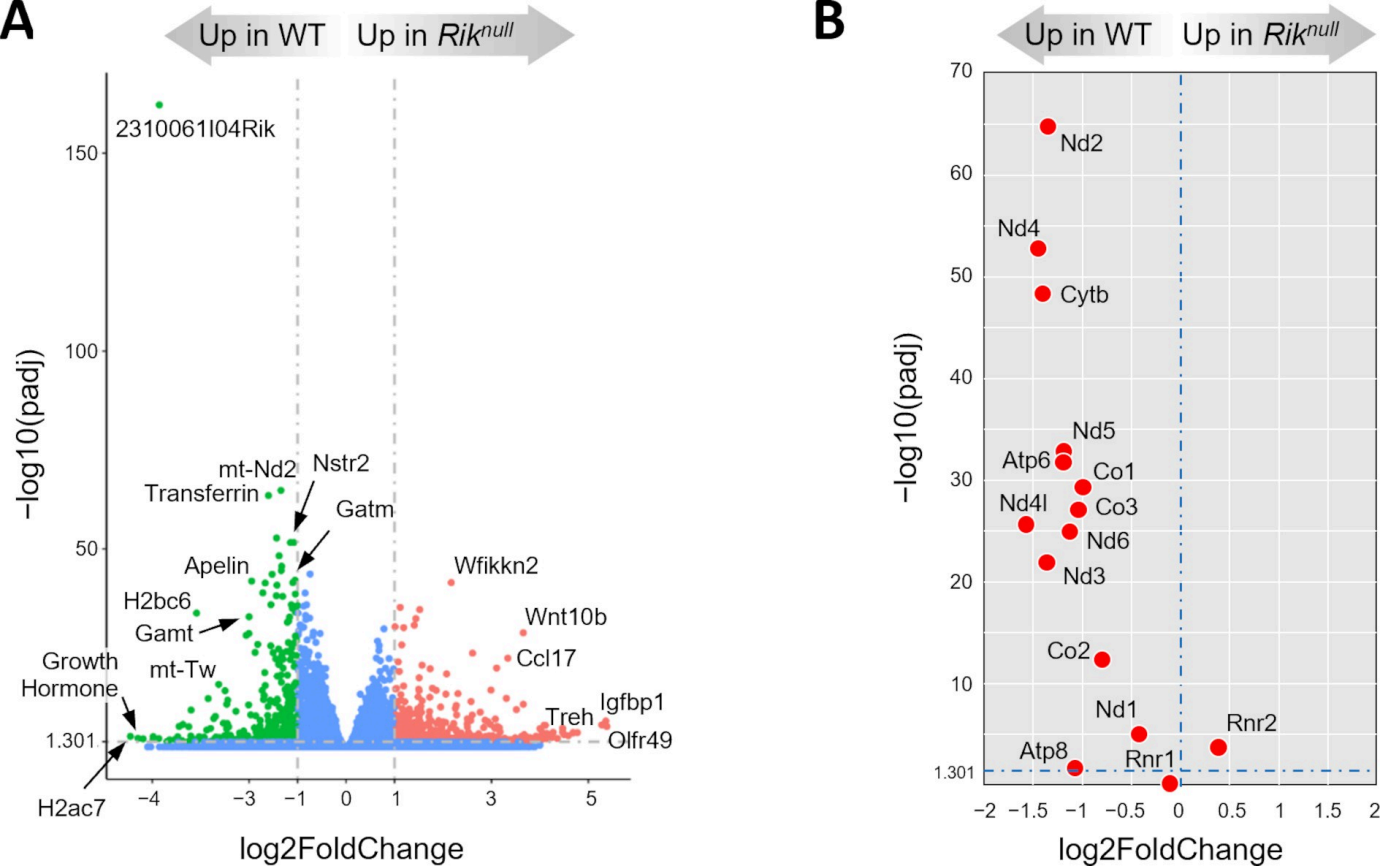
Analysis of 23 days old *Rik*^*null*^ brain transcriptome. (**A**) Volcano plot of RNA-seq analysis visualizing significant DEGs in P23 *Rik*^*null*^ versus WT (control) brains. Magnitude of change (x-axis) vs. adjusted *p* values (padj) (y-axis). Points that have a fold change less than 2 (log_2_ = 1) or have a *padj* value less than 0.05 (-log10 = 1.301) are shown in blue (n = 6 WT, n = 8 *Rik*^*null*^). (**B**) Scatter plot of mitochondrial gene expression levels for 13 protein-coding and 2 ribosomal RNA genes: *Rik*^*null*^ versus WT relative expression (x-axis) vs. statistical significance (y-axis) of the difference.

Next, we compared transcriptomes of WT and *Rik*^*null*^ brains at P23, the peak of the difference between the groups. RNA-seq analysis of P23 *Rik*^*null*^ brains revealed that the groups were well separated on PCA plot (S5A Fig). There were 452 and 601 DEGs expressed in WT or *Rik*^*null*^ brains, respectively (S5B Fig). Heat map analysis demonstrated high sample similarity within each group and a clear separation between the groups (S5C Fig). As expected, *Rik* showed the greatest difference in relative mRNA expression between WT and *Rik*^*null*^ brains (Fig 3A). All mitochondrial messenger RNAs exhibited significant enrichment in WT brains compared to *Rik*^*null*^ brains at P23 (Fig 3B), consistent with results at P14. Similarly, *mt-Rnr2* displayed increased expression in *Rik*^*null*^ brains compared to WT controls, while the expression of *mt-Rnr1* did not differ between groups. Interestingly, *transferrin* (*Trf*), which encodes a critical iron transporter, displayed one of the highest differences in expression between *Rik*^*null*^ WT control brains (Fig 3A). *Rik*^*null*^ brains also expressed lower levels of genes of two enzymes responsible for biosynthesis of creatine, *glycine amidinotransferas*e (*Gatm*) and *guanidinoacetate methyltransferase* (*Gamt*). Of note, oligodendrocytes (OLs) exhibit the highest average level of both *Gatm* and *Gamt* mRNA expression across the entire body [21]. KEGG pathway enrichment analysis revealed reduced expression of several genes associated with neurodegenerative pathways in *Rik*^*null*^ brains (S6A Fig). In contrast, there was elevated expression of genes associated with the cytokine-cytokine receptor interaction pathway in *Rik*^*null*^ brains (S6B Fig).

**Fig 4:**
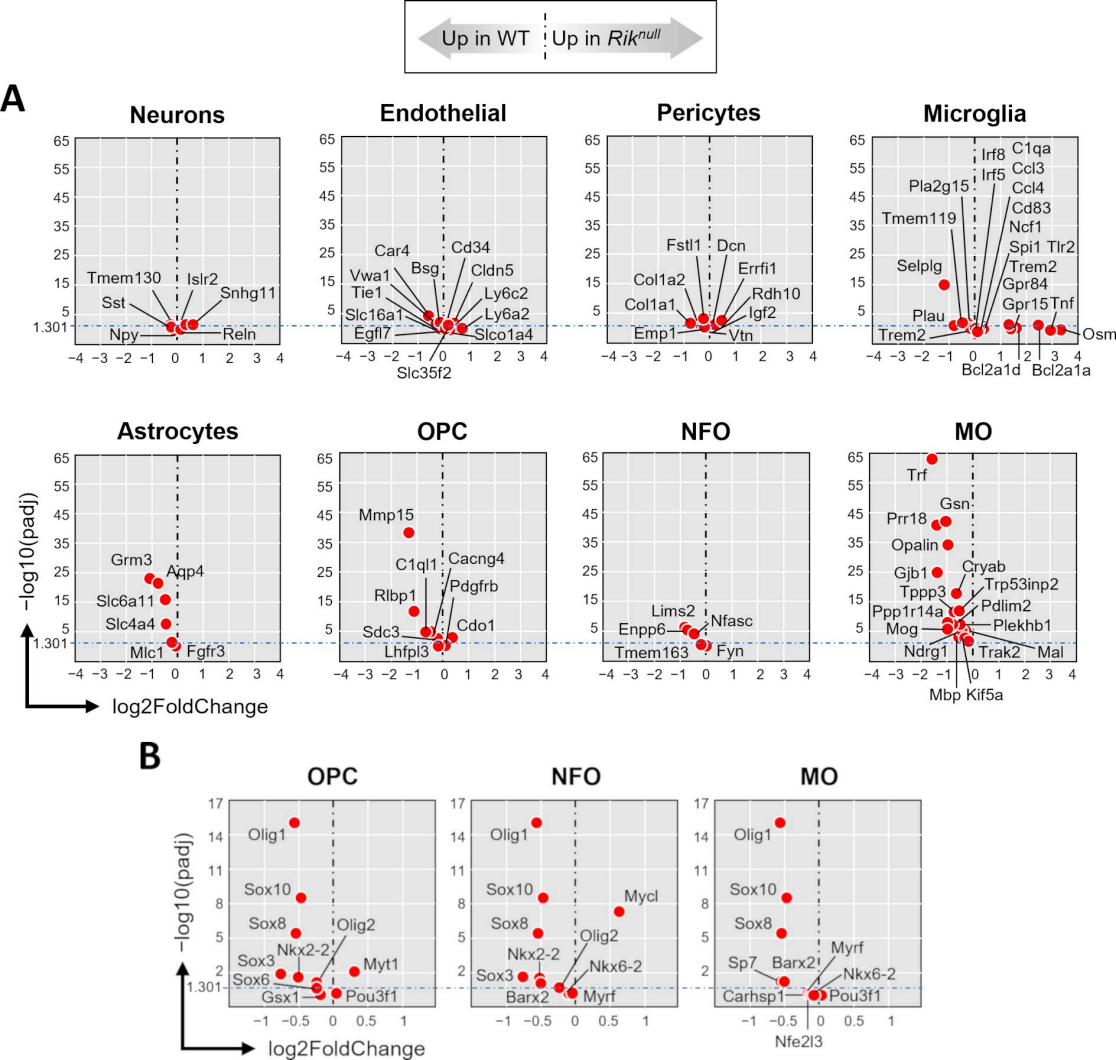
Low expression of genes specific for myelinating oligodendrocytes in 23 days old *Rik*^*null*^ mouse brains. Scatter plots of mRNA expression levels of gene markers specific for different cell populations in in P23 *Rik*^*null*^ versus WT mouse brains from RNA-seq DE analysis: Relative expression (x-axis) vs. statistical significance (y-axis) of difference in mRNA expression. OPC: Oligodendrocyte precursor cells; NFO: Newly formed oligodendrocytes; MO: Myelinating oligodendrocytes. (**A**) Cell lineage-specific marker genes. (**B**) Marker genes coding transcription factors in oligodendrocyte populations.

To ascertain whether the differences in transcriptome profiles between *Rik*^*null*^ and WT brains could be attributed to specific cell populations, we compared the expression of the most highly expressed genes (excluding transcription factors) specific to several key cell populations in the brain [22]. The analysis revealed no significant differences in the expression of the selected neuronal (*Islr2*, *Npy*, *Reln*, *Snhg11*, *Sst*, *Tmem10*), endothelial (*Bsg*, *Car4*, *Cd34*, *Cldn5*, *Egfl7*, *Ly6a2*, *Ly6c2*, *Slc16a1*, *Slc35f2*, *Slco1a4*, *Tie1*, *Vwa1*), and pericyte (*Col1a1*, *Col1a2*, *Dcn*, *Emp1*, *Errfi1*, *Fstl1*, *Igf2*, *Rdh10*, *Vtn*) marker genes (Fig 4A and S3 and S4 Files). The only microglia-specific DEG showing significantly lower expression in *Rik*^*null*^ brains compared to WT brain was *selectin*, *platelet (p-selectin) ligand* (*Selplg*, also called *CD162*), suggesting that microglia were relatively unaffected in the *Rik*^*null*^ brain. The non-significant microglia DEGs were: *Bcl2a1a*, *Bcl2a1d*, *C1qa*, *Ccl3*, *Ccl4*, *Cd83*, *Gdf15*, *Gpr84*, *Irf5*, *Irf8*, *Ncf1*, *Osm*, *Pla2g15*, *Plau*, *Spi1*, *Tlr2*, *Tmem119*, *Tnf*, *and Trem2*. Four out of six astrocyte-specific genes displayed lower expression in *Rik*^*null*^ brains compared to WT brains. These genes included *aquaporin 4* (*Aqp4*) and *glutamate receptor*, *metabotropic 3* (*Grm3*), suggesting dissimilar astrocyte transcriptome profiles in *Rik*^*null*^ and WT brains, as well as *Slc4a4* and *Slc6a11*, while *Fgfr3* and *Mlc1* did not differ significantly. Interestingly, OL precursor cells (OPCs) had two significant DEGs, *Mmp15* and *Rlbp1*, while the expression of two other well-known OPC marker genes, *platelet-derived growth factor receptor*, *alpha polypeptide (Pdgfra*) and *chondroitin sulfate proteoglycan 4* (*Cspg4*, also called *Ng2*) [23], did not differ between *Rik*^*null*^ and WT brains (Fig 4A and S4 File). Other OPC gene DEGs included: *Cdo1*, *C1ql1*, *Lhfpl3*, *Mmp15*, and *Sdc3*. Gene expression by newly formed OLs (NFOs) did not differ between *Rik*^*null*^ and WT brains; these genes included *Enpp6*, *Fyn*, *Lims2*, *Nfasc*, and *Tmem163*. In contrast, six genes (*Cryab*, *Gjb1*, *Gsn*, *Opalin*, *Prr18*, *Trf*) specific to myelinating OLs (MOs) showed lower expression in *Rik*^*null*^ brains compared to WT brains, suggesting potential dysfunction in MOs. Importantly, these DEGs included *Opalin*, a gene encoding a CNS myelin marker specifically expressed in MOs that is important for fine-tuning of exploratory behavior [24]. Other non-significant DEGs included: *Kif5a*, *Mbp*, *Mog*, *Ndrg1*, *Pdlim2*, *Plekhb1*, *Ppp1r14a*, *Tppp3*, *Trak2*, and *Trp53inp2*.

Next, we compared the expression of oligodendrocyte-specific transcription factors in *Rik*^*null*^ and WT brains [22]. Pan-OL transcription factors *oligodendrocyte transcription factor 1* (*Olig1*), and *SRY (sex determining region Y)-box transcription factors* (*Sox8* and *Sox10*) differed significantly and showed lower expression in *Rik*^*null*^ OLs compared to WT OLs (Fig 4B and S5 File). In contrast, the expression of *Olig2* (expressed only in OPC and NFO populations) did not differ significantly between *Rik*^*null*^ and WT brains. Other non-significant OPC DEGs were: *Gsc1*, *Myt1*, *Nkx2-2*, *Pou3fl*, *Sox3*, and *Sox6*. Moreover, *myelin regulatory factor* (*Myrf*) (expressed only in NFO and MO populations) was expressed to the same extent in *Rik*^*null*^ and WT brains. Non-significant NFO DEGs included: *Barx2*, *Mycl*, *Nkx2-2*, *Nkx6-2*, and *Sox3*. Non-significant MO DEGs included: *Barx2*, *Carhsp1*, *Nfe2l3*, *Nkx6-2*, *Pou3f1*, and *Sp7*. Taken together, these results suggest that *Rik* is potentially important for differentiation of NFOs into MOs in the mouse brain during the third week of postnatal development.

### Discreet myelination of anterior thalamic nuclei in P23 *Rik*^*null*^ brains

**Fig 5:**
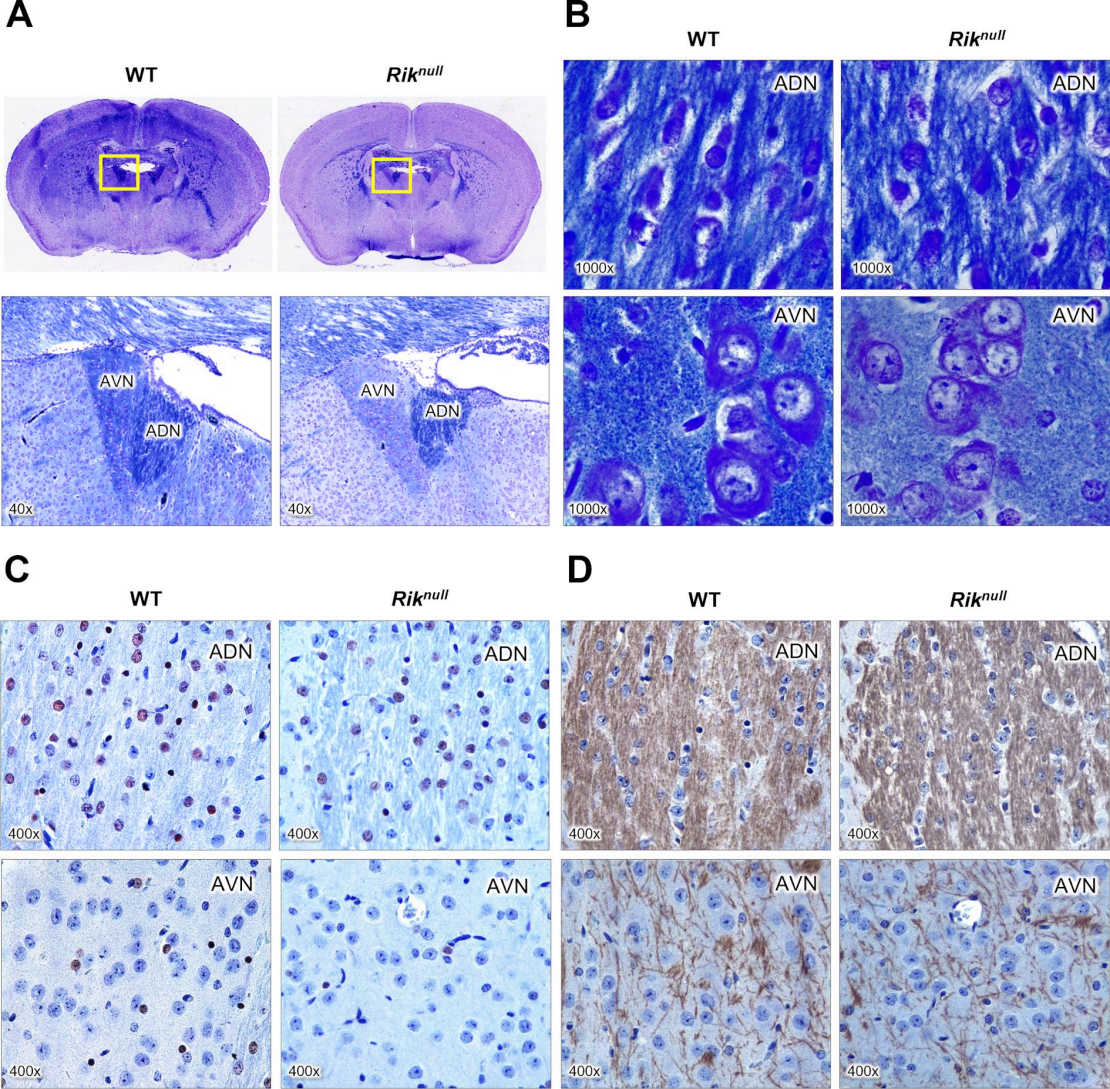
RIK deficiency causes defects in myelinated tracts of the anterior thalamus. (**A**) Luxol Fast Blue (LFB) staining of brain coronal sections from 23 days old WT and *Rik*^*null*^ mice. Top panels are representatives of brain sections at Bregma −0.58 mm. Bottom panels show magnified images of the area within yellow windows above containing anteroventral nuclei (AVN) and anterodorsal nuclei (ADN) of the thalamus. (**B**) Representative views of LFB-stained ADN and AVN at higher magnification as marked. (**C** and **D**) Hematoxylin and Eosin (H & E) and immunohistochemistry staining of ADN and AVN nuclei with oligodendrocyte-specific anti-Olig2 (C) and anti-MBP (D) antibodies. Brown color indicates corresponding protein location. Cell nuclei are blue.

Under visual examination, the brain of P23 mutant mice appeared without gross deformities, except that whole brain, and particularly the cortical hemispheres, appeared consistently smaller compared to WT controls (S7A Fig). To understand the consequences of *Rik* deficiency for CNS myelination, we used the myelin stain Luxol Fast Blue (LFB) to stain *Rik*^*null*^ brains. Of note, LFB stains myelinated fibers in bright blue, Nissl substance in dark blue, and cell nuclei in blue. As shown, the LFB staining intensity in *Rik*^*null*^ brain sections was reduced compared to WT sections (top panels in Fig 5A), suggesting decreased total myelination of *Rik*^*null*^ brains compared to their WT counterparts. The anterior thalamic nucleus (ATN), containing the anterodorsal nuclei (ADN) and the anteroventral nuclei (AVN), was one of the most impacted regions (bottom panels in Fig 5A). Although staining intensity of the ADN in *Rik*^*null*^ and WT brains was similar, LFB intensity in the AVN in *Rik*^*null*^ appeared much weaker compared WT controls. At higher magnification, *Rik*^*null*^ and WT and demonstrated similar intensity of longitudinal axon fibers (top panels in Fig 5B). In contrast, cross-sectioned axons in *Rik*^*null*^ AVN displayed decreased LFB-staining intensity compared to WT counterparts (bottom panels in Fig 5B). Moreover, the cytoplasm of *Rik*^*null*^ cells appeared much smaller and contained less Nissl substance, suggesting decreased protein biosynthesis compared to WT OLs. Hematoxylin and Eosin staining (H&E) of those regions demonstrated no obvious variations in cellularity or general structure between mutant and control ATNs (S7B Fig). The images from Fig 5 represent one mouse per genotype so we were not able to reliably quantify the number of OLs or the overall intensity of the MBP staining, which is a limitation of this study. Since this quantification would provide additional evidence for the myelination defects, we will ensure a more rigorous evaluation in our future studies.

### Decreased myelination of anterior commissure (AC) in P23 *Rik*^*null*^ brains

**Fig 6:**
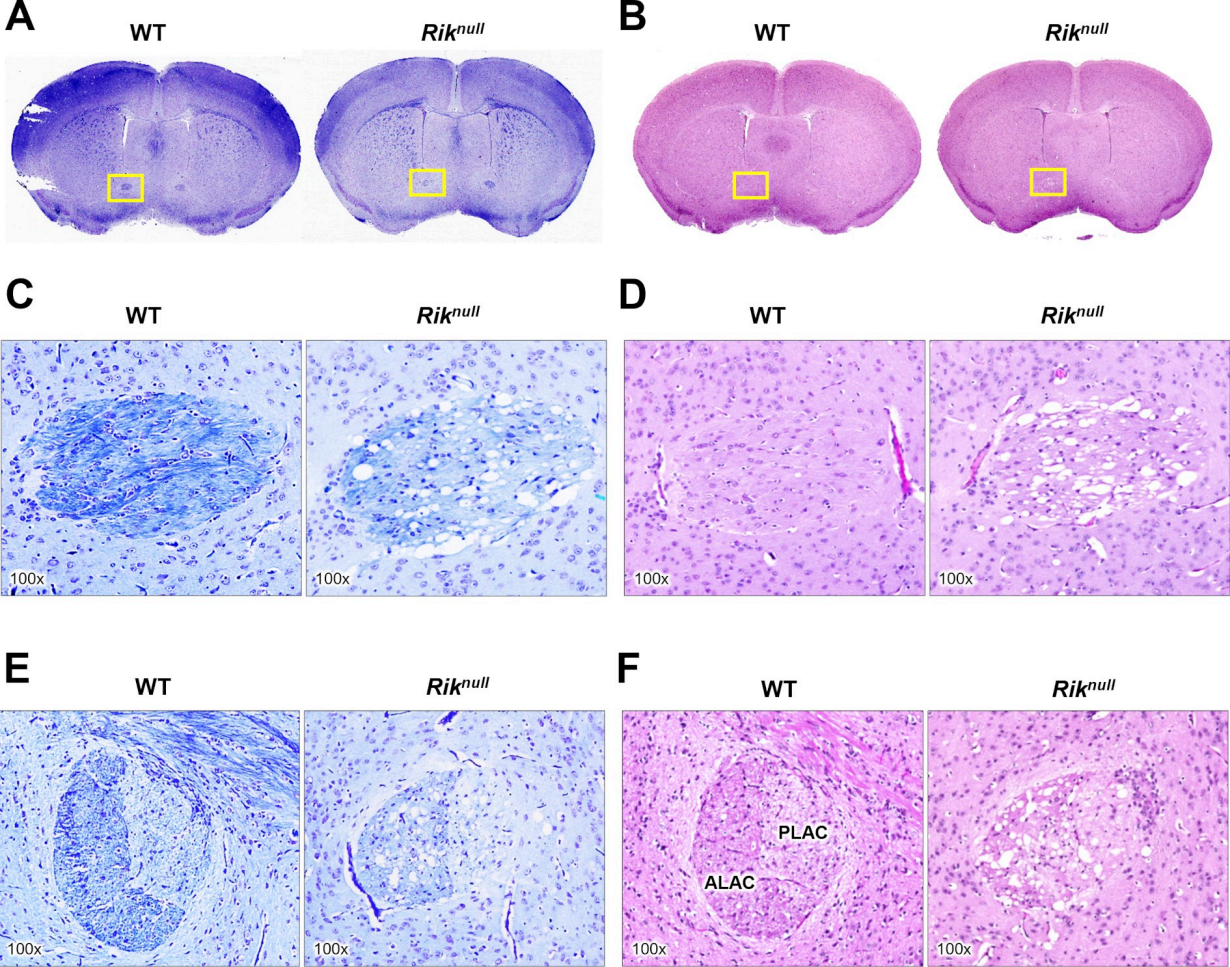
RIK deficiency causes defects in myelinated tracts of anterior commissure. Histological investigation of brain coronal sections from 23 days old mice at Bregma 0.38 mm. WT and *Rik*^*null*^ brain sections were stained with LFB (A and C), H&E (B and D). (**C** and **D**) Insets of yellow windows in panels (**A** and **B**) above at higher magnification. (**E** and **F**) Magnified image of anterior commissure on sagittal sections (not shown here) from P23 mice (~lateral 0.24 mm). ALAC: Anterior limb of the anterior commissure; PLAC: Posterior limb of the anterior commissure. Total magnification of images is marked on each image.

Further analysis revealed dramatic divergence in myelination in the AC of WT and *Rik*^*null*^ brains in AC (Fig 6). *Rik*^*null*^ AC displayed much weaker LFB staining compared to WT AC. The difference was apparent in both coronal and sagittal views (Fig 6A and 6C). Similar to other mouse models with myelination defects [25,26], numerous vacuoles of varying size were detected in AC of *Rik*^*null*^ mice, but not in WT mice (Fig 6A and 6C) (Table 1). The extensive vacuolation was also evident with H&E staining (Fig 6B and 6D).

**Table 1:** Quantification of vacuolation area in the anterior commissure (AC) of Rik^null^ mice.

	Area of vacuolation (μm^2^)				
Animal #	≤ 100 μm^2^	101–200 μm^2^	201–300 μm^2^	≥ 301 μm^2^	Average area (μm^2^)
**1**	**69.2**	**146.7**	**246.0**	**499.8**	**204.0**
**2**	**63.8**	**125.9**	**251.2**	**490.0**	**176.4**
**3**	**63.4**	**136.0**	**241.1**	**478.8**	**164.9**
**Mean**	**65.5**	**136.2**	**246.1**	**488.2**	**181.8**
**SD**	**3.3**	**10.4**	**5.0**	**12.6**	**20.1**
	**Number of vacuoles by size (% of total vacuoles)**				
**Animal #**	**n (% of total)**	**n (% of total)**	**n (% of total)**	**n (% of total)**	**Average n (% of total)**
**1**	**35 (37.2%)**	**26 (27.7%)**	**14 (14.9%)**	**19 (20.2%)**	**94 (100%)**
**2**	**39 (47.0%)**	**16 (19.3%)**	**15 (18.1%)**	**13 (15.7%)**	**83 (100%)**
**3**	**53 (54.1%)**	**17 (17.3%)**	**12 (12.2%)**	**16 (16.3%)**	**98 (100%)**
**Mean**	**42.3 (46.1%)**	**19.7 (21.4%)**	**13.7 (15.1%)**	**16.0 (17.4%)**	**91.7 (100%)**
**SD**	**9.5 (8.5%)**	**5.5 (5.5%)**	**1.5 (2.9%)**	**3.0 (2.5%)**	**7.8 (0%)**

**Fig 7:**
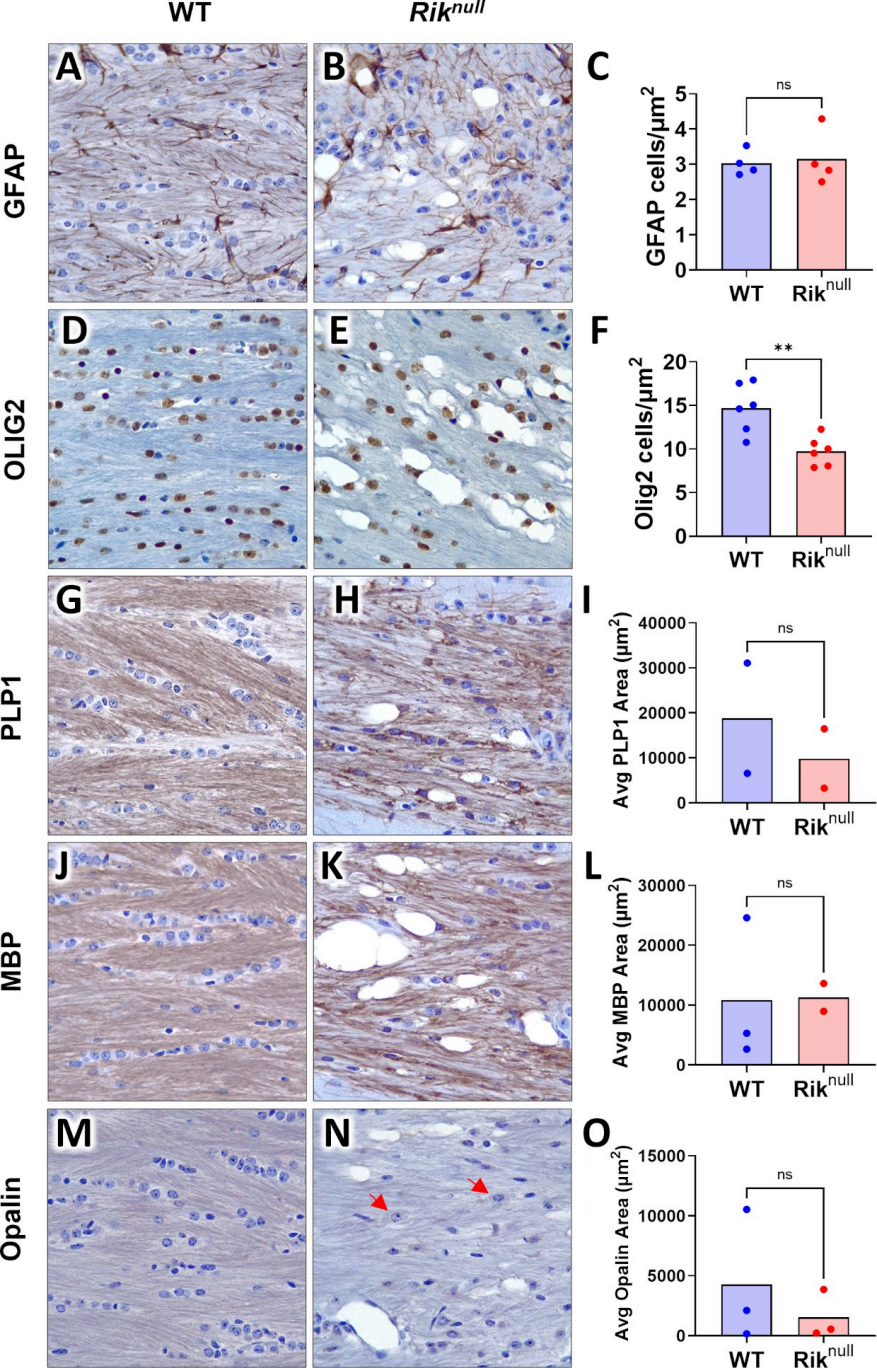
Immunohistochemical (IHC) analysis of 23 days old WT and *Rik*^*null*^ mouse brains. Representative IHC with hematoxylin staining of WT and *Rik*^*null*^ mouse brains at P23 with anti-GFAP antibody, an astrocyte-specific marker (**A** and **B**) or antibodies against the oligodendrocyte-specific markers Olig2 (**D** and **E**), PLP1 (**G** and **H**), MBP (**J** and **K**), and Opalin (**M** and **N**). Quantification of immunoreactive cells for each marker: GFAP (**C**),Olig2 (**F**), PLP1 (**I**), MBP (**L**), and Opalin (**O**) (n = 2–6 animals/genotype). Scatter plots indicate mean value. Total magnification of all images is 400x. ** p<0.01 unpaired Student’s *t* test, ns—non-significant. Regions of coronal sections at Bregma −0.58 mm show parts of anterior commissure heavily myelinated in WT as shown in Fig 6C.

To assess the potential cellular basis for the histological observations, we stained for cell lineage specific markers. Staining with antibodies to glial fibrillary acidic protein (GFAP), a major marker of mature astrocytes in CNS, showed the expected astrocytic morphology in the *Rik*^*null*^ brain (Fig 7A and 7B), suggesting no obvious abnormalities in astrocyte development, in agreement with our RNA-seq results (Fig 4A). However, the ramification of fine processes of *Rik*^*null*^ astrocytes looked more disorganized compared to WT counterparts. While a rigorous assessment of astrocyte morphology was not conducted, we did find that the number of GFAP immunoreactive cells was the same between WT and *Rik*^*null*^ ACs (Fig 7C, *t*_*6*_ = 0.295, p = 0.778, unpaired Student’s *t* test). Staining for OLIG2 revealed a significant decrease in OLIG2 positive cells in *Rik*^*null*^ compared to WT mice (Fig 7F, *t*_*10*_ = 3.721, p = 0.004, unpaired Student’s *t* test), with the distribution of *Rik*^*null*^ OLs looking more irregular compared to WT counterparts, which mostly aligned along the axonal fibers (Fig 7D and 7E). Immunolabeling for proteolipid protein 1 (PLP1) and myelin basic protein (MBP) showed a reduction of myelin in the AC of *Rik*^*null*^ mice compared to controls (Fig 7G, 7H, 7J and 7K). There was a trend for a decrease in the average area of immunoreactivity for PLP1 (Fig 7I, *t*_*2*_ = 0.642, p = 0.587, unpaired Student’s *t* test) while MBP area was not different (Fig 7L, *t*_*3*_ = 0.049, p = 0.964, unpaired Student’s *t* test) in *Rik*^*null*^ compared to WT mice. Mature OLs and their myelin sheaths, identified by Opalin immunolabeling (Fig 7M and 7N), appeared to show more intense labeling in the WT AC than in the AC of *Rik*^*null*^ mice (Fig 7O, *t*_*4*_ = 0.803, p = 0.467, unpaired Student’s *t* test). Interestingly, the intensity of staining of OL cytoplasm in AC of *Rik*^*null*^ mice was stronger than the myelin sheaths.

## Discussion

In this study, we demonstrate that a 59-bp homozygous deletion in the first intron of the *2310061I04Rik* gene abolishes its expression. Mouse *Rik* is predicted to encode five protein isoforms of different length with a 315 amino acid long canonical isoform (Uniprot.org entry #B8JJ69). RIK protein is evolutionarily conserved from *C*. *elegans* to mammals (https://useast.ensembl.org/Homo_sapiens/Gene/Compara_Tree?db=core;g=ENSG00000204564;r=6:30647039–30653210). The human orthologue of RIK, C6orf136, shows altered expression in various solid tissue cancers [27]. Mammalian RIK proteins consist of two domains: a proline-rich domain and a domain of unknown function 2358 (duf2358). RIK has no recognized function. However, it is probably unique since there are no RIK paralogues in the human or mouse genome. RIK localizes to mitochondria (https://personal.broadinstitute.org/scalvo/MitoCarta3.0/mouse.mitocarta3.0.html), indicating that RIK might be important for mitochondria activity.

When the *Rik*^*null*^ phenotype was first observed on the *Traf7*^*fl/fl*^ background (S1 Fig), the weight of runt *Traf7*^fl*/fl*^ mice reached a maximum of 7g, and they died before P40. *Rik*^*null*^ mice carrying wildtype *Traf7* after backcrossing into C57BL6/J strain reached the same weight, but the majority of them died before P25, substantially earlier than runt *Traf7*^fl*/fl*^ mice. These discrepancies in life expectancy between mutant *Rik*^*null*^ and *Traf7*^fl*/fl*^ mice with *Rik* deletion may be due to complex multigenic differences among commonly used C57BL/6 substrains [13,14]. These differences also may result from differences among genes located between *Rik* and the centromere of the telocentric chromosome 17 (Fig 1A), which includes the whole *Qki* locus. Interestingly, in addition to its role regulating processing of MBP mRNA, *Qki* has recently been shown to directly affect the regulation of cholesterol biosynthesis necessary for myelinogenesis [28,29].

The human and mouse mitochondrial genomes are a single circular double-stranded DNA molecule of almost 17 kilobase pairs [30,31]. MtDNA contains 37 genes that encode 13 proteins, 2 ribosomal RNAs, and 22 transfer RNAs. Our RNA-seq experiments demonstrated low expression of most mitochondrial genes in P14 and P23 *Rik*^*null*^ brains. Of the 15 genes, encoding messenger and ribosomal RNAs, 14 showed reduced expression, while only *mt-Rnr2* demonstrated increased expression in *Rik*^*null*^ brains compared to WT controls (Figs 2 and 3). On the other hand, mtDNA copy numbers were increased in *Rik*^*null*^ brains compared to controls (Fig 2C), indicating that *mt-Rrn2* was the only gene expressed in agreement with the mtDNA copy number in *Rik*^*null*^ brains, while the lower expression of other mitochondrial messenger RNAs and *mt-Rrn1* contradicted the higher mtDNA copy numbers in *Rik*^*null*^ brains. Since all 15 mitochondrial genes, except *mt-ND6*, are transcribed from the same heavy strand promoter, these observations suggest that RIK may regulate the stability of mitochondrial RNAs. Further evaluation of RIK effects on mitochondrial function, *Rik*^*null*^ mouse brains showed lower expression of genes encoding subunits of all electron transport pumps, including respiratory complex I (*mt-Nd2*, *mt-Nd4* and others), complex III (*mt-Cyb*), and complex IV (*mt-Co1*). It is interesting that, similar to *Rik*^*null*^ animals, mice deficient in a subunit of mitochondrial complex I, *NADH*:*ubiquinone oxidoreductase core subunit S4* (*Ndufs4*), which is a model of Leigh syndrome [32], were born healthy [33], but by P21, they lagged behind in growth, developed ataxia and died prematurely. However, important differences between *Ndufs4* and *Rik*^*null*^ mice also were noted, including that *Ndufs4* knockout mice reached almost twice the body weight (15g at P28) and survived almost twice as long (up to P50) as *Rik*^*null*^ mice. Thus, even though both mouse models demonstrate mitochondrial defects and runting, the differences in lifespan and body size between *Ndufs4* knockout and *Rik*^*null*^ mice may result from the different background strains for each mouse. Future studies are needed to delineate molecular mechanisms of RIK on mitochondrial function.

Based on the RNAseq (Fig 4) and immunohistochemical studies (Figs 5–7), we next evaluated oligodendrogenesis in *Rik*^*null*^ mice. At P23, *Rik*^*null*^ brains demonstrated lower expression of *Gatm* and *Gamt*, which function together to synthesize creatine, which facilitates recycling of ATP by converting ADP back to ATP. Mature OLs are the major producers of creatine during postnatal development [34] and express higher levels of both *Gatm* and *Gamt* than any other brain cell type, including OPCs [21]. Although *Gatm-* and *Gamt-*deficient mice consistently weigh less than control littermates, they survive until 24 weeks of age and beyond [34–36]. Overall, the phenotype of *Gatm-* or *Gamt-*deficient mice is much milder compared to *Rik*^*null*^ mice. Therefore, a decreased expression of *Gatm* and *Gamt* in P23 *Rik*^*null*^ brains suggests that RIK may either directly control their gene expression, or indirectly affect their expression by regulating OL maturation.

P23 *Rik*^*null*^ brains expressed almost 4-fold lower levels of *Trf*, which encodes transferrin, compared to WT controls. Transferrin is a major transporter of iron, which is an essential nutrient for myelin production, and serves as a cofactor for enzymes involved in ATP production, and cholesterol and lipid biosynthesis. Furthermore, OLs show the highest iron concentrations in the brain, which is directly linked to their elevated metabolism associated with the high-energy process of myelination [37,38]. To be transported through blood and across OL cellular membranes, iron binds to transferrin [39,40], which is essential for OL maturation and function [41,42]. Interestingly, OLs are most vulnerable to transferrin deficiency during the premyelinating stage [43].

It is noteworthy that the genes specific for mature OLs, including *Trf*, *Gatm*, *Gamt*, and *Opalin*, displayed no differences in their expression between P14 *Rik*^*null*^ and WT brains, while the expression of mitochondrial genes was already reduced at that time. Considering this, we propose that RIK is important for optimal transcription of mitochondrial genes, specifically in the brain during the third week of postnatal development, when the phenotypic divergence of OL maturation between *Rik*^*null*^ and control mice occurs. During this period of postnatal development, the most rapid phase of myelination begins in the mouse brain [23,44–46] and the highest supply of cellular energy is required [47]. Because our investigation of brains from *Rik*^*null*^ mice revealed that RIK deficiency disturbed the generation of mature OL, we tentatively propose to name RIK as an “oligodendrocyte maturation factor” (OMF).

## Materials and methods

### Discovery of Rik^null^ mice

All housing and experimental use of mice were carried out in AAALAC-accredited facility in accordance with United States federal, state, local, and institutional regulations and guidelines governing the use of animals and were approved by OUHSC Institutional Animal Care and Use Committee. The *Rik*^*null*^ allele arose as a spontaneous recessive mutation in the *2310061I04Rik* gene during generation of conditional *Traf7* knockout mice. The *Traf7* targeting vector and *Traf7*^*+/fl*^ mice were generated by the Ingenious Targeting Laboratory (Ronkonkoma, NY, USA) on the C57BL/6 background. Since *Rik* is located in proximity of *TRAF7*^*fl*^ allele on chr17, we unlinked *TRAF7*^*fl*^ and *Rik*^*null*^ alleles by crossbreeding of *Traf7*^+/fl*^Rik^*+/Null*^ littermates until we obtained several mice without pathology. The resulting *Rik*^*null/+*^ mice were backcrossed on the C56BL/6 background for several generations and were genotyped to confirm presence of *Rik*^*null*^ allele and absence of *TRAF7*^*fl*^ allele. Mice of both sexes were used for all assays. Mice were humanely euthanized by CO_2_ asphyxiation immediately prior to tissue collection.

### Tissue DNA preparation

For DNA preparation tissue biopsy was incubated overnight with 0.1 mg/mL proteinase K (Fisher Scientific, Hampton, NH, USA) in ATL buffer at 55°C. Proteins and lipids were precipitated with 1/3 of volume of 5M NaCl and debris was removed by centrifugation at 14,000 rpm for 10 minutes. DNA was precipitated with 7/10 of volume of 100% isopropanol and centrifugation for 10 minutes at 14,000 rpm. The pellet was washed in 70% ethanol and dissolved in TE. DNA concentration was measured with Nanodrop (Fisher Scientific).

### Mouse genotyping

Genotyping of tail DNA was performed on purified genomic DNA using conventional and/or real-time PCR. Genotyping of *TRAF7*^*fl*^ allele was described in Tsitsikov et. al. [15]. Conventional PCR with primers mRik-Fwd: AGATGGAGAAGTCGGTGGA and mRik-Rev: TTTCTCCCATGGAGCAGTAAAC yield amplifies a 401 bp DNA fragment from WT and a 342 bp fragment from 2310061I04Rik^Null^ alleles, respectively. Real-time PCR was performed as a DuPlex reaction for both alleles in one tube using PerfeCTa® qPCR FastMix® II, Low ROX™ (VWR, Radnor, PA, USA, Quanta Biosciences™, #95120) on an Applied Biosystems 7500 Fast Real-Time PCR System (Fisher Scientific). TaqMan Assays for WT and Null alleles are as follows:

WT-Fwd: AGAAATCAGCTCATGCTCCTC
WT-Rev: GTCTCCCTTTCCTCTGGTAAAC
WT-Probe: AGCTGGGCTGATCAGAAGCTTCAA (HEX-BHQ1)
Null-Fwd: AGGGCTACACAGACCTAGTTT
Null-Rev: CTCTTTCCTCATGTCTCCCTTTC
Null-Probe: ACCAAACAAAGGAACCTGCAGAAGAGA (FAM-BHQ1)

### RNA extraction

Mouse brains were collected and stored in Invitrogen™ RNAlater™ Stabilization Solution (Fisher Scientific, AM7023) before RNA extraction. Total RNA was purified using the RNeasy Lipid Tissue Mini Kit (QIAGEN, Redwood City, CA, USA, 74804) according to the manufacturer’s instructions, quantified by NanoDrop and stored at -80°C.

### RNA-seq and differential expression (DE) analysis

Total brain RNA from at least six WT and 2310061I04RikNull mouse littermates of either sex was used for RNA-seq experiments. Preparation of cDNA libraries, sequencing, and Standard bioinformatics analyses were conducted by Novogene Co., LTD (Beijing, China). Significant DEGs were defined as those that had both an absolute log2FoldChange ≥ 1 as well as a false discovery rate adjusted p-value ≤ 0.05 for each comparison independently.

### Histology

Brains from mice (littermates, different ages, either sex) were fixed in 4% paraformaldehyde, paraffin embedded, and sectioned at 4 μM using standard methods. Sections were stained with either hematoxylin and eosin using Abcam H&E Staining Kit (Fisher Scientific, NC1881153) or Luxol Fast Blue (LFB) using the StatLab LUXOL FAST BLUE KIT (Fisher Scientific, NC9030259) for myelin. Staining was performed according to the manufacturers’ instructions. Images were obtained at total magnifications of 40x and 100x (combination of magnifications of 4x and 10x objective lens with 10x ocular lens) using a Leica (Wetzlar, Germany) DM750 microscope with an ICC50 W Camera Module and included software. Up to seven sections from mice of each genotype were evaluated for each genotype.

### Immunohistochemistry

Slides were deparaffinized in three changes of xylene, two changes of 100% alcohol, two changes of 95% alcohol, and rehydrated in two changes of H_2_O. Next, slides were bleached with 3% Hydrogen Peroxide (Sigma-Aldrich, St. Louis, Mo, USA, #516813) and masked epitopes were recovered with eBioscience™ IHC Antigen Retrieval Solution—High pH (10X) (Invitrogen, Waltham, MA, USA, #00-4956-58). Slides were blocked for 1 hour at room temperature with blocking solution (1xPBS, 3% milk, 5% Normal Goat Serum, 0.1% Triton X-100, 0.01% NaN3) and incubated with antibodies overnight at 4°C. The antibody dilutions were as follows: GFAP (D1F4Q) XP Rabbit mAb (Cell signaling, Danvers, MA, USA, #12389) (1:500); PLP1 (E9V1N) Rabbit mAb (Cell signaling #28702) (1:1000); MBP (D8X3Q) XP Rabbit mAb (Cell signaling #78896) (1:2000); Opalin polyclonal rabbit antibody (Sigma-Aldrich #HPA014372) (1:1000) and Olig2 polyclonal rabbit antibody (Sigma-Aldrich, #AB9610) (1:200). Slides were washed in three changes of 1xPBS and incubated in SignalStain® Boost Detection Reagent (HRP, Rabbit, Cell signaling #8114) for 1 hour at room temperature. Bound antibodies were revealed with Metal Enhanced DAB Substrate Kit (Thermo fisher, Waltham, MA, USA, #34065). Slides were counterstained with Hematoxylin for 2 minutes and Bluing Reagent for 15 seconds. Images were quantified as the average number of positive cells per square micrometer, average immunoreactivity area in square micrometers, or vacuolation area in square micrometers from 40x-400x field(s) per region of interest for a brain area, one-six animals per genotype using ImageJ (NIH v1.54).

### Mitochondrial copy number (mtDNA-CN) measurement

The mtDNA-CN was determined using a duplexed real time qPCR ΔΔC_T_ assay. The cycle threshold (C_T_) value of three mitochondrial genes (*Atp6*, *Rnr2*, and *Nd4*) and nuclear-specific (*Gapdh*) target genes were determined in several replicates for each sample. Relative measure of mtDNA-CN is reported as the difference in C_T_ values (ΔC_T_) for each pair of genes. The mtDNA-CN is presented as relative quantities (RQ) of mitochondrial DNA and calculated by comparing ΔC_T_ of samples from *Rik*^*null*^ and WT mouse tissues. Assays were run as described in Mouse genotyping section above. TaqMan Assays for *Gapdh* and three mitochondrial genes are as follows:

CN_mGAPDH-Fwd TCCTCAGTGTAGCCCAAGA
CN_mGAPDH-Rev CAGTGACTTGGGACAAGGATAG
CN_mGAPDH-Probe TGCCTGCTTCACCACCTTCTTGAT (HEX/BHQ-1)
CN_m_mtATP6-Fwd CAGGCTTCCGACACAAACTA
CN_m_mtATP6-Rev TGTAAGCCGGACTGCTAATG
CN_m_mtATP6-Probe TCACTTGCCCACTTCCTTCCACAA (FAM/BHQ-1)
CN_m_mtRNR2-Fwd GGACATCCCAATGGTGTAGAA
CN_m_mtRNR2-Rev AGATAGAAACCGACCTGGATTG
CN_m_mtRNR2-Probe CCTACGTGATCTGAGTTCAGACCGGA (FAM/BHQ-1)
CN_m_mtND4-Fwd GCCTCACATCATCACTCCTATT
CN_m_mtND4-Rev GGCTATAAGTGGGAAGACCATT
CN_m_mtND4-Probe TGCCTAGCAAACTCCAACTACGAACG (FAM/BHQ-1)

### Statistics

Samples sizes were based on previous studies and limited by availability of genotypes in each litter. GraphPad Prism 9.0 was used for analysis. Data was presented as median±SEM, mean±SD, or scatter plots indicating mean value. P < 0.05 was considered statistically significant. Two-factor analysis of variance (ANOVA) with Bonferroni post-hoc analysis was used for Fig 2C. Unpaired Student’s *t* test was used for immunohistochemical quantification. In RNA-seq DE analysis, differential expression was calculated using the Wald test implemented in the R package DESeq2. *mtDNA-CN* were calculated by comparative ΔC_T_ experiment runs on AB7500 Fast machine and analyzed using the 7500 Software v2.3. Calculations of *mtDNA-CN* were performed by Excel with the built-in analysis methods. ImageJ 1.54 was used to quantify immunoreactivity and to measure vacuolation area.

## Supporting information

S1 Fig Severe runting and a shortened life-span of *Traf7*^*fl/fl*^ mice with serendipitous deletion in *2310061I04Rik* gene (*Traf7*^fl*/fl*^). (**A**) Mouse weight chart from P13 to end of life of *Traf7*^fl*/fl*^ mice. (n = 391 (1–20 animals) WT, n = 481 (1–30 animals) *Traf7*^+/fl*^, n = 230 (1–15 animals) *Traf7*^fl*/fl*^). Data presented as median±SEM. (**B**) Representative 4-weeks old WT and *Traf7*^fl*/fl*^ littermates. (**C**) Probability of survival of *Traf7*^fl*/fl*^ mice. n = 25 *Traf7*^fl*/fl*^.
(TIF)

S2 Fig Differential gene expression in WT and *Traf7*^fl*/fl*^ mouse embryos at E9.5. (**A**) PCA Plot of RNA-seq analysis in WT and *Traf7*^fl*/fl*^ mouse embryos. Each point corresponds to an individual embryo. (**B**) Venn diagram of RNA-seq analysis in WT and *Traf7*^fl*/fl*^ mouse embryos showing DEGs overlap. (**C**) Heatmap of mRNA expression levels for all significant DEGs in WT and *Traf7*^fl*/fl*^ mouse embryos. (**D**) Volcano plot of RNA-seq analysis visualizing significant DEGs in WT versus *Traf7*^fl*/fl*^ embryos: magnitude of change (*x-axis*) vs. statistically significant *p* values (*y*-axis). Points with *p* value less than 0.05 (-log10 = 1.301) are shown in blue.
(TIF)

S3 Fig Differential gene expression in 14 days old WT and *Rik*^*null*^ brains. (**A**) PCA Plot of RNA-seq analysis in WT and *Rik*^*null*^ brains. Each point corresponds to an individual brain sample. (**B**) Venn diagram of RNA-seq analysis in *Rik*^*null*^ versus WT brains showing DEGs overlap. (**C**) Heatmap of mRNA expression levels for all significant DEGs in *Rik*^*null*^ versus WT brains.
(TIF)

S4 Fig KEGG enrichment analysis of signaling pathways in P14 *Rik*^*null*^ brains. Signaling pathways upregulated in WT versus *Rik*^*null*^ brains (A) or in *Rik*^*null*^ versus WT brains (B). Each bubble represents a KEGG pathway. Gene ratio (x-axis) is the proportion of the total genes in a given pathway that is upregulated in the indicated group.
(TIF)

S5 Fig Differential gene expression in 23 days old WT and *Rik*^*null*^ brains. (**A**) PCA Plot of RNA-seq analysis in WT and *Rik*^*null*^ brains. Each point corresponds to an individual brain sample. (**B**) Venn diagram of RNA-seq analysis in *Rik*^*null*^ versus WT brains showing DEGs overlap. (**C**) Heatmap of mRNA expression levels for all significant DEGs in *Rik*^*null*^ versus WT brains.
(TIF)

S6 Fig KEGG enrichment analysis of signaling pathways in 23 days old *Rik*^*null*^ brains. Signaling pathways upregulated in WT versus *Rik*^*null*^ brains (A) or in *Rik*^*null*^ versus WT brains (B). Each bubble represents a KEGG pathway. Gene ratio (x-axis) is the proportion of the total genes in a given pathway that is upregulated in the indicated group.
(TIF)

S7 Fig Morphological comparison of WT and *Rik*^*null*^ brains. (**A**) Images of a dorsal view of the brains from 3 weeks old WT and *Rik*^*null*^ littermates. (**B**) Coronal sections of brain shown in (A) stained with hematoxylin and eosin. Top panels are representatives of brain sections at about Bregma −0.58 mm. Bottom panels show magnified images of the area within black windows above containing anteroventral nuclei (AVN) and anterodorsal nuclei (ADN) of the thalamus.
(TIF)

S8 Fig Images analyzed for Table 1. (TIF)

S9 Fig Uncropped DNA gel used for Fig 1B. (TIF)

S10 Fig Images analyzed for Fig 7. (TIF)

S1 File E9.5 *TRAF7*^*KO*^-asterisk vs. WT DEGs. (XLSX)

S2 File P14 *Rik*^*null*^ vs. WT DEGs. (XLSX)

S3 File P23 *Rik*^*null*^ vs. WT DEGs. (XLSX)

S4 File Differentially expressed genes in brain cell populations P23 *Rik*^*null*^ vs. WT for Fig 4A. (XLSX)

S5 File Differentially expressed genes in brain cell populations P23 *Rik*^*null*^ vs. WT for Fig 4B. (XLSX)

S6 File Raw data for figures. (XLSX)

S7 File Movie 1 TRAF7^fl*/fl*^ vs. WT. (MP4)

S8 File Movie 2 Rik^null^ vs. WT. (MP4)
