# Supplementary figures and images for: Spontaneous mutation in 2310061I04Rik results in reduced expression of mitochondrial genes and impaired brain myelination
Source: PLoS One. 2024 Dec 4;19(12):e0290487. doi: 10.1371/journal.pone.0290487 (PMC11617004; doi:10.1371/journal.pone.0290487)

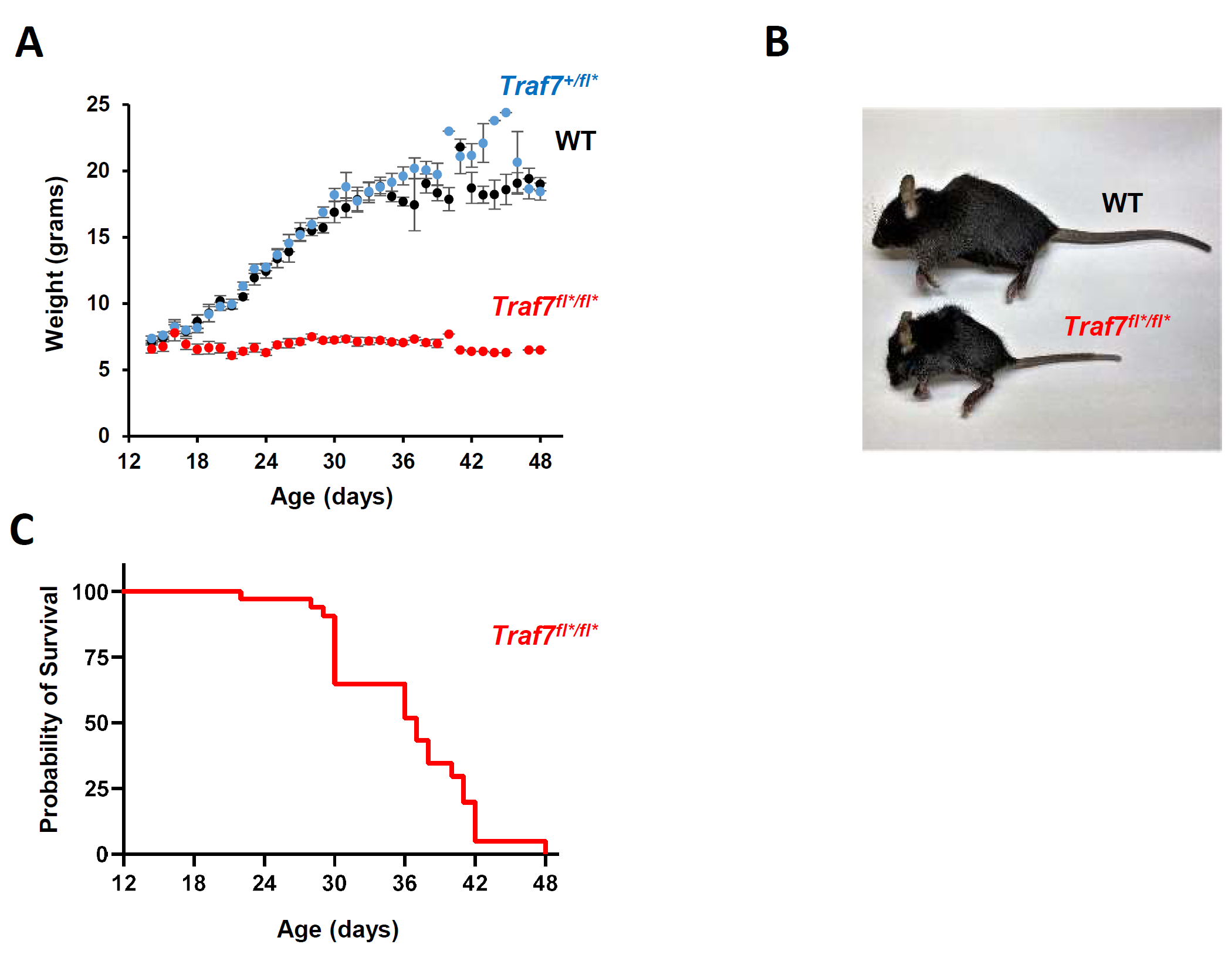

Supplement: S1 Fig — (A) Mouse weight chart from P13 to end of life of Traf7fl*/fl* mice. (n = 391 (1–20 animals) WT, n = 481 (1–30 animals) Traf7+/fl*, n = 230 (1–15 animals) Traf7fl*/fl*). Data presented as median±SEM. (B) Representative 4-weeks old WT and Traf7fl*/fl* littermates. (C) Probability of survival of Traf7fl*/fl* mice. n = 25 Traf7fl*/fl*. (TIF) [file pone.0290487.s001.tif]

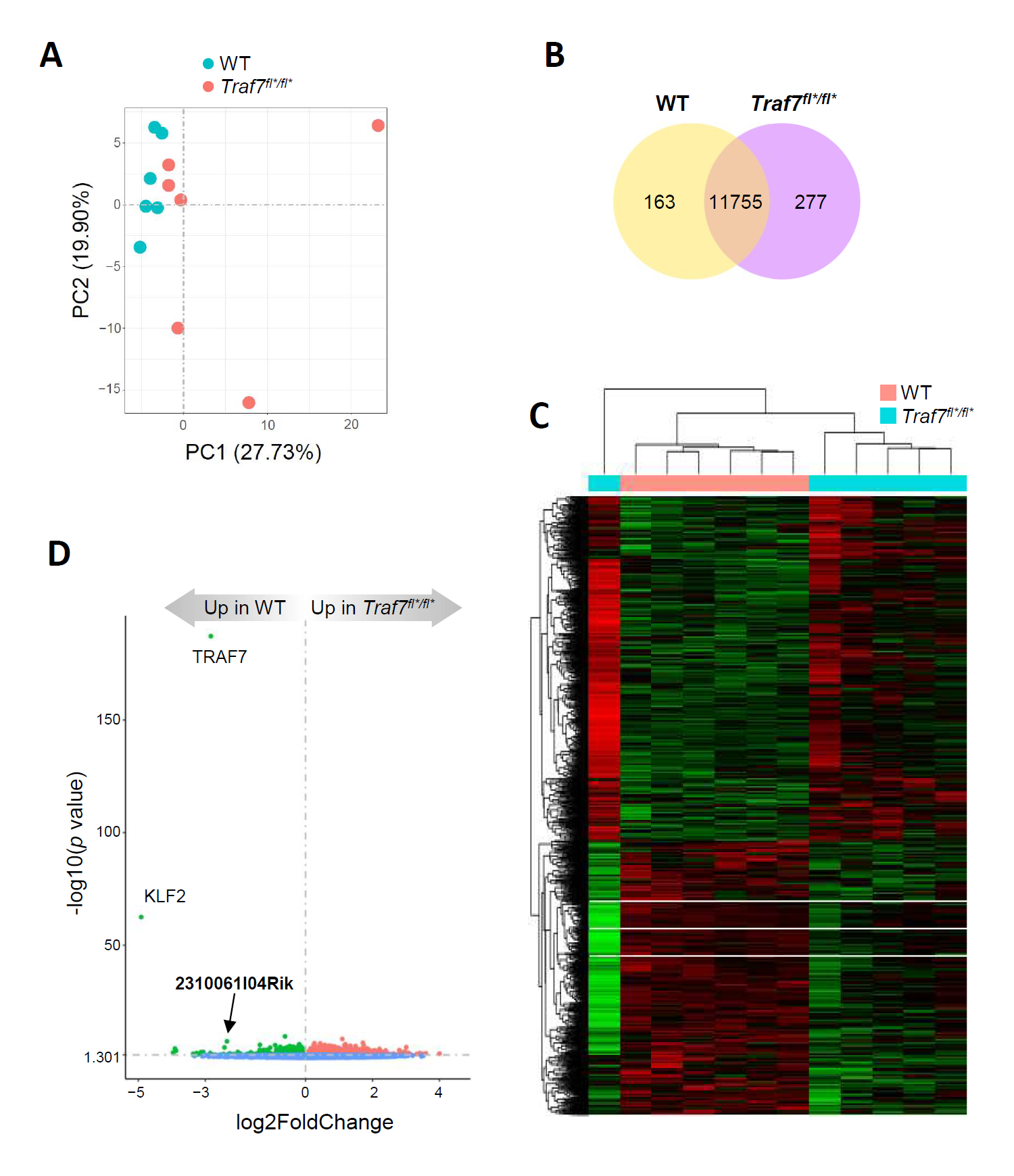

Supplement: S2 Fig — (A) PCA Plot of RNA-seq analysis in WT and Traf7fl*/fl* mouse embryos. Each point corresponds to an individual embryo. (B) Venn diagram of RNA-seq analysis in WT and Traf7fl*/fl* mouse embryos showing DEGs overlap. (C) Heatmap of mRNA expression levels for all significant DEGs in WT and Traf7fl*/fl* mouse embryos. (D) Volcano plot of RNA-seq analysis visualizing significant DEGs in WT versus Traf7fl*/fl* embryos: magnitude of change (x-axis) vs. statistically significant p values (y-axis). Points with p value less than 0.05 (-log10 = 1.301) are shown in blue. (TIF) [file pone.0290487.s002.tif]

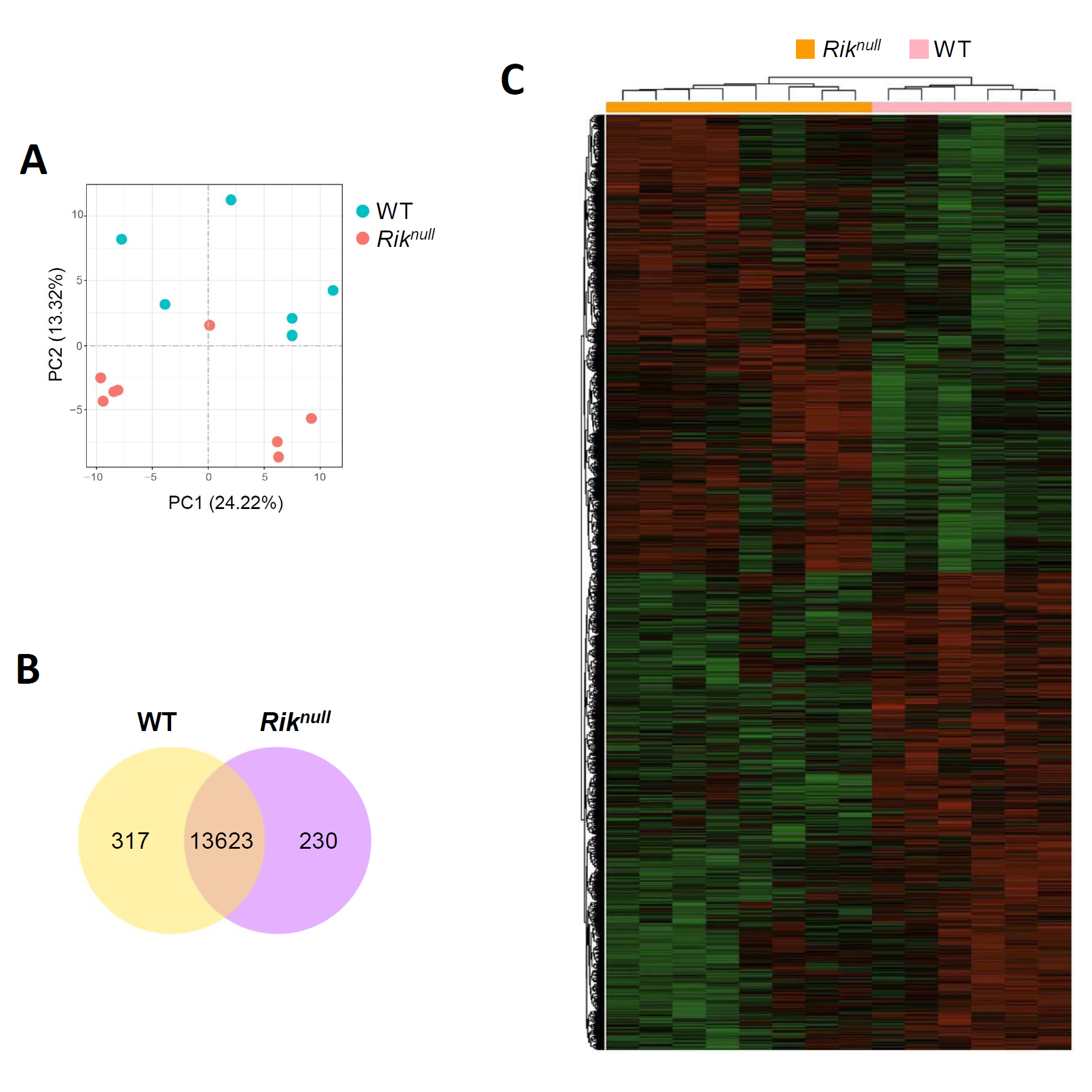

Supplement: S3 Fig — (A) PCA Plot of RNA-seq analysis in WT and Riknull brains. Each point corresponds to an individual brain sample. (B) Venn diagram of RNA-seq analysis in Riknull versus WT brains showing DEGs overlap. (C) Heatmap of mRNA expression levels for all significant DEGs in Riknull versus WT brains. (TIF) [file pone.0290487.s003.tif]

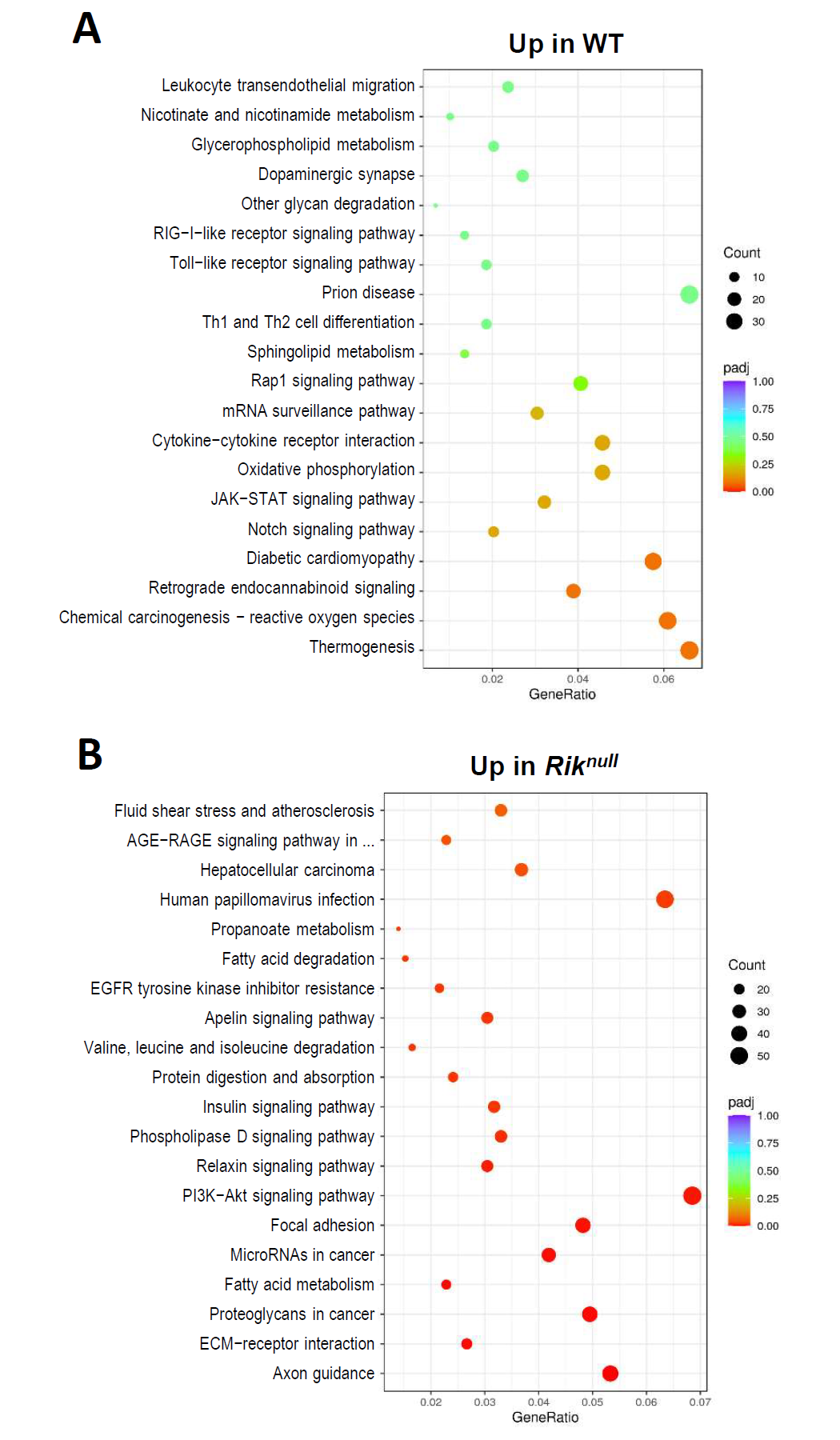

Supplement: S4 Fig — Signaling pathways upregulated in WT versus Riknull brains (A) or in Riknull versus WT brains (B). Each bubble represents a KEGG pathway. Gene ratio (x-axis) is the proportion of the total genes in a given pathway that is upregulated in the indicated group. (TIF) [file pone.0290487.s004.tif]

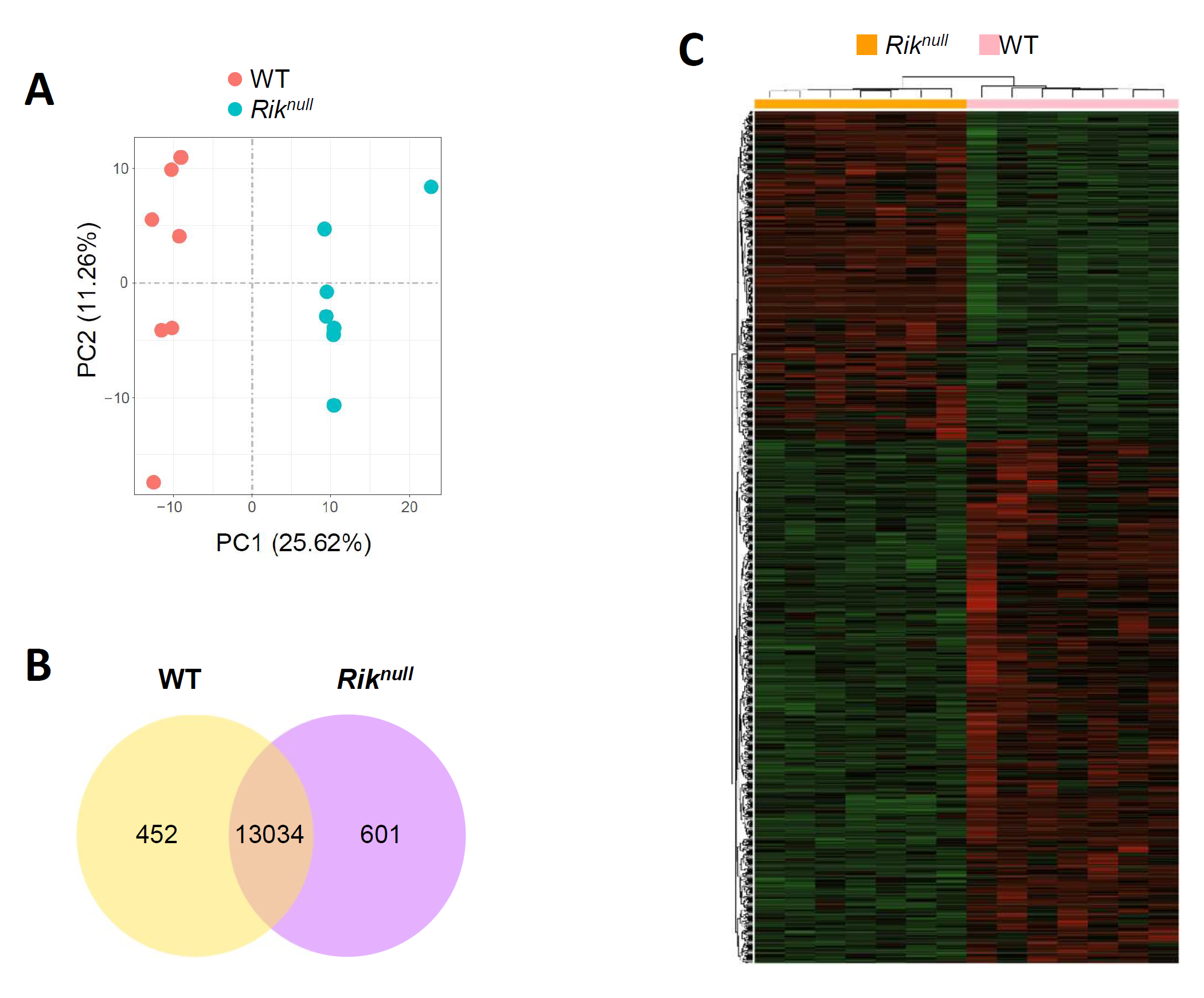

Supplement: S5 Fig — (A) PCA Plot of RNA-seq analysis in WT and Riknull brains. Each point corresponds to an individual brain sample. (B) Venn diagram of RNA-seq analysis in Riknull versus WT brains showing DEGs overlap. (C) Heatmap of mRNA expression levels for all significant DEGs in Riknull versus WT brains. (TIF) [file pone.0290487.s005.tif]

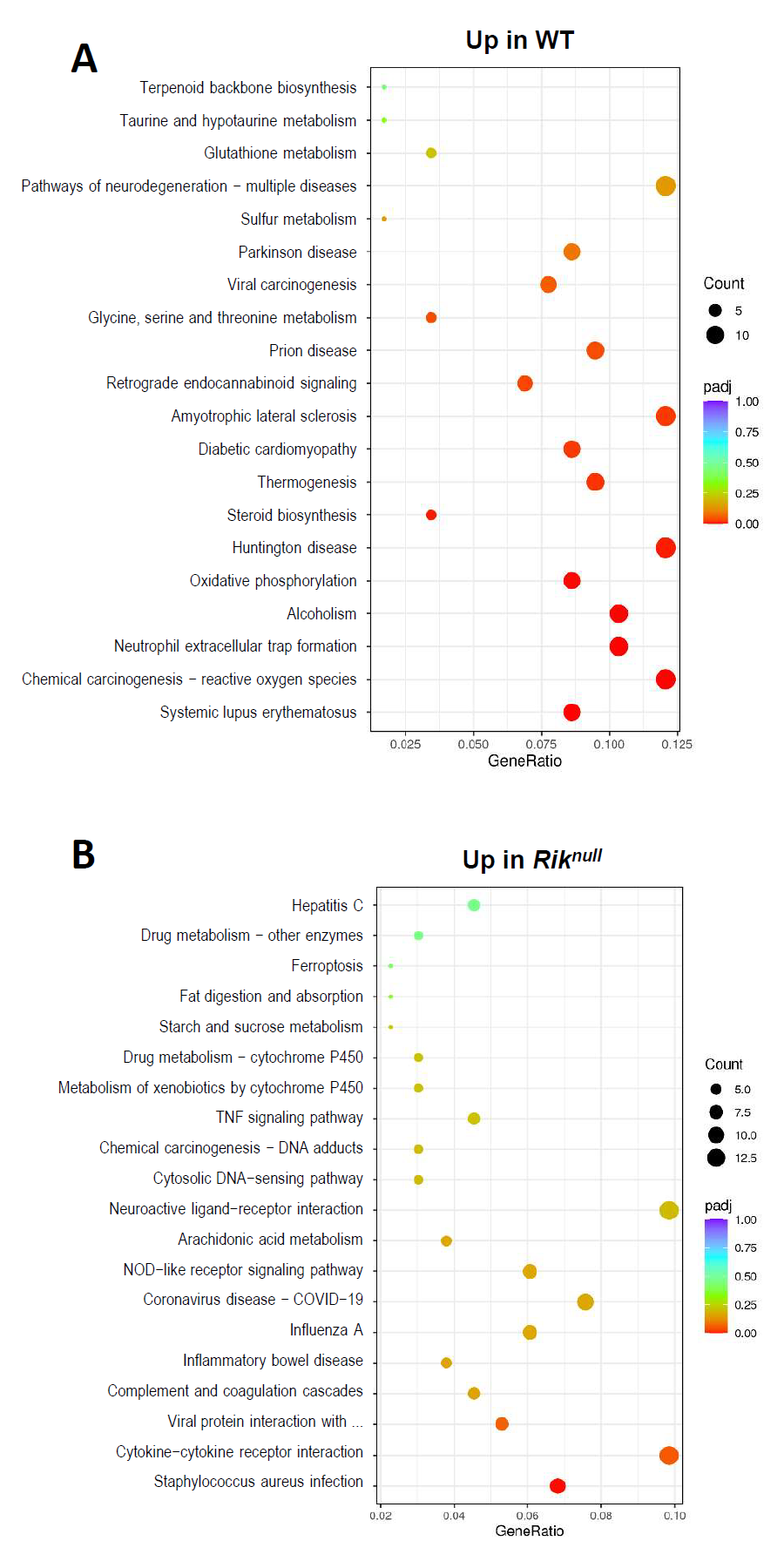

Supplement: S6 Fig — Signaling pathways upregulated in WT versus Riknull brains (A) or in Riknull versus WT brains (B). Each bubble represents a KEGG pathway. Gene ratio (x-axis) is the proportion of the total genes in a given pathway that is upregulated in the indicated group. (TIF) [file pone.0290487.s006.tif]

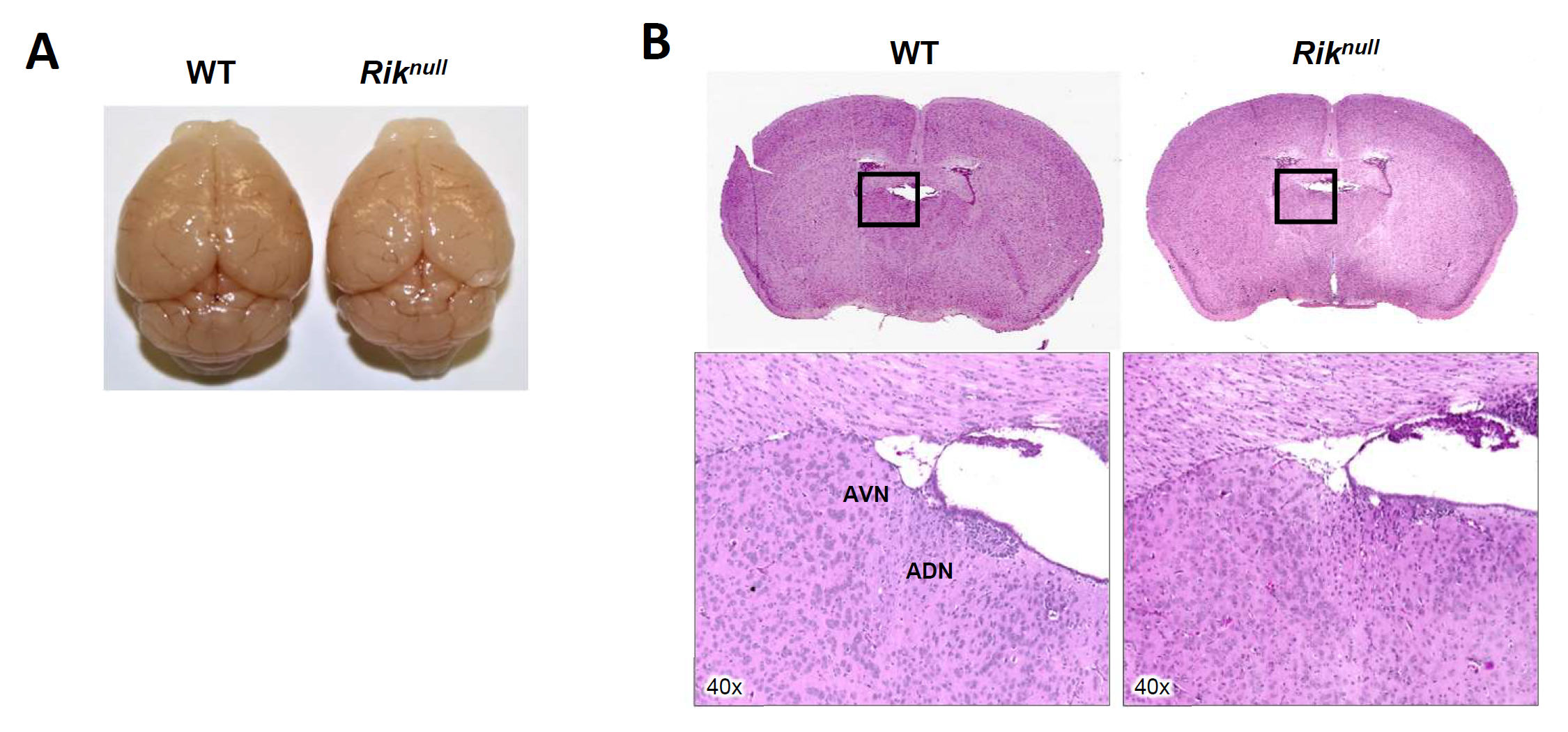

Supplement: S7 Fig — (A) Images of a dorsal view of the brains from 3 weeks old WT and Riknull littermates. (B) Coronal sections of brain shown in (A) stained with hematoxylin and eosin. Top panels are representatives of brain sections at about Bregma −0.58 mm. Bottom panels show magnified images of the area within black windows above containing anteroventral nuclei (AVN) and anterodorsal nuclei (ADN) of the thalamus. (TIF) [file pone.0290487.s007.tif]

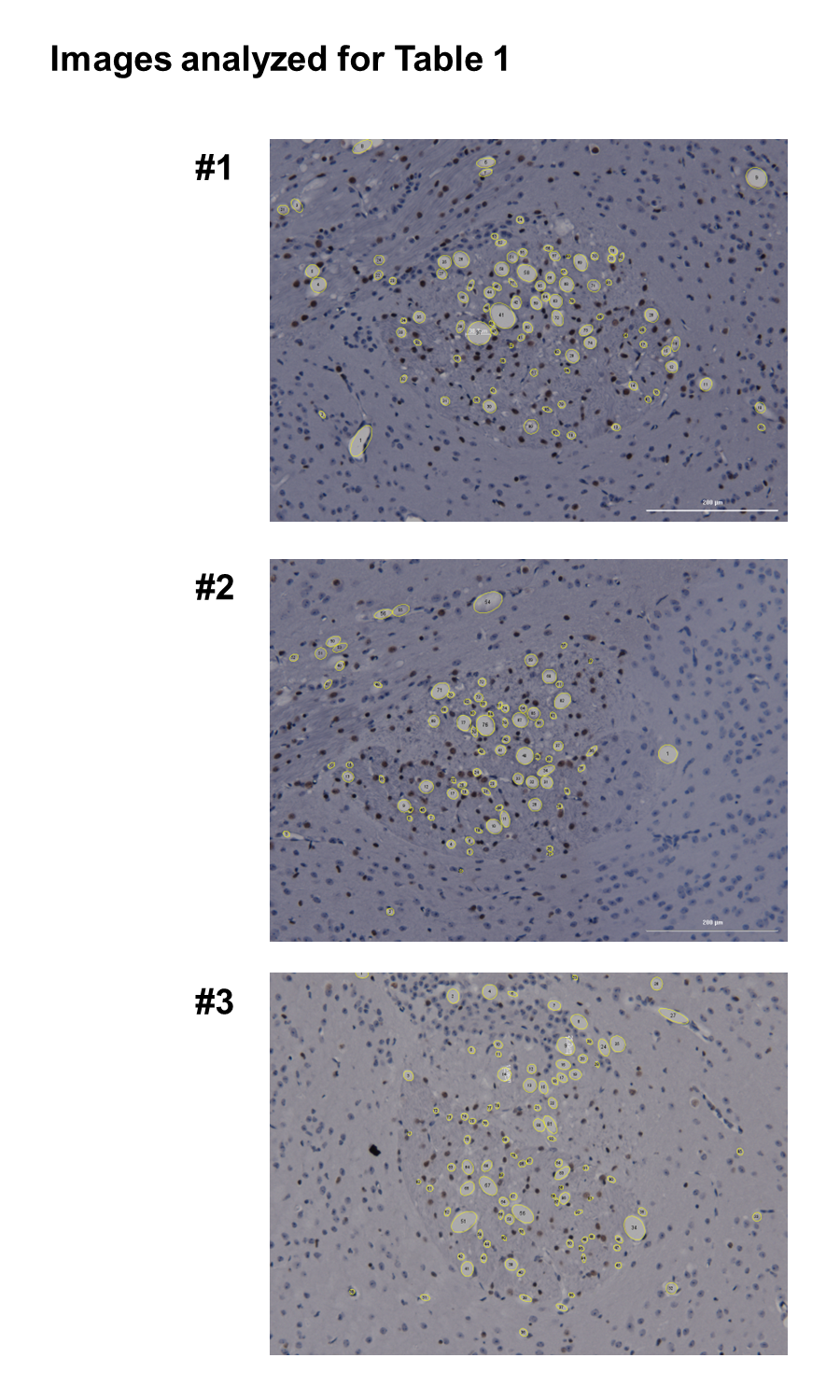

Supplement: S8 Fig — (TIF) [file pone.0290487.s008.tif]

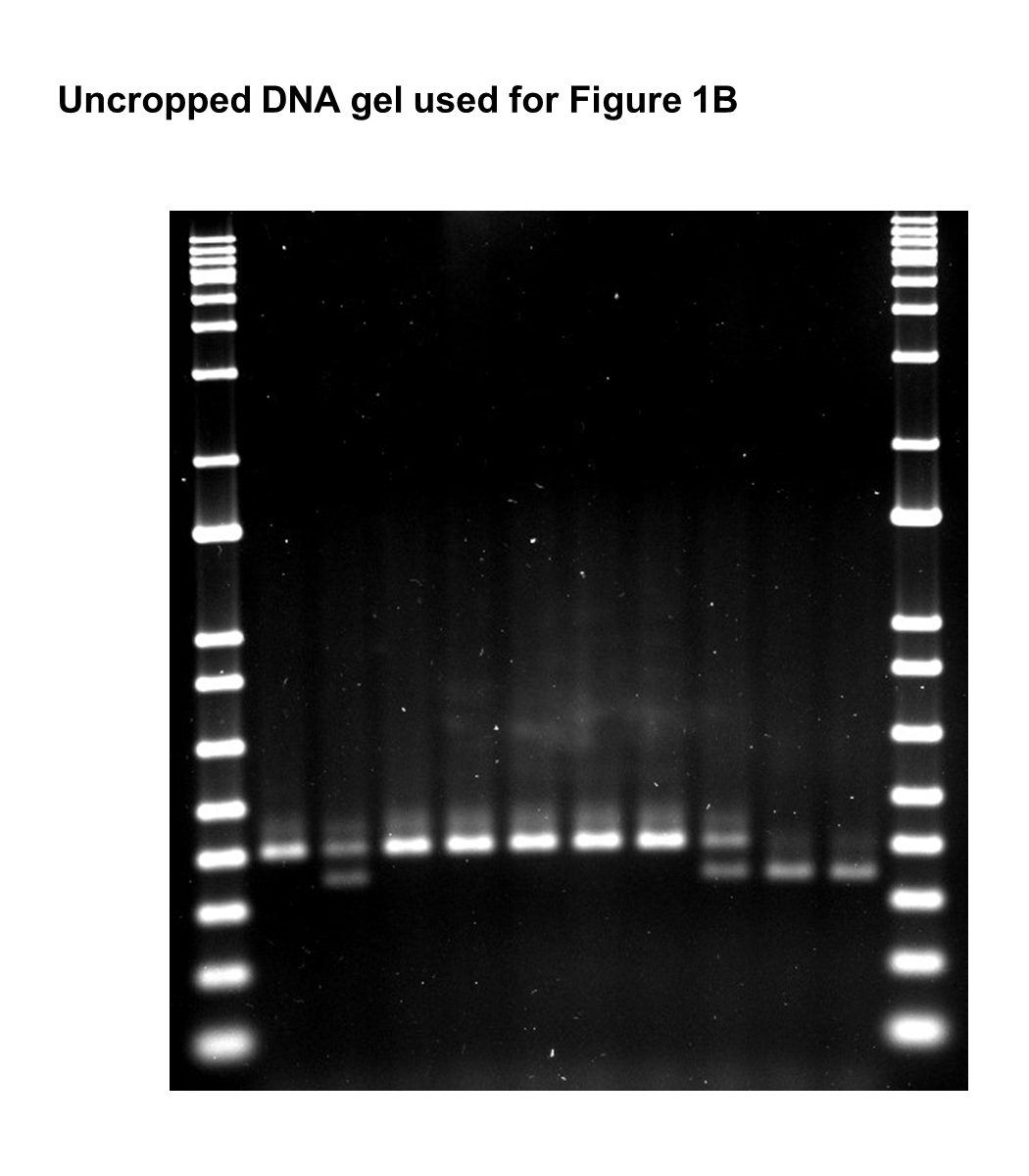

Supplement: S9 Fig — (TIF) [file pone.0290487.s009.tif]

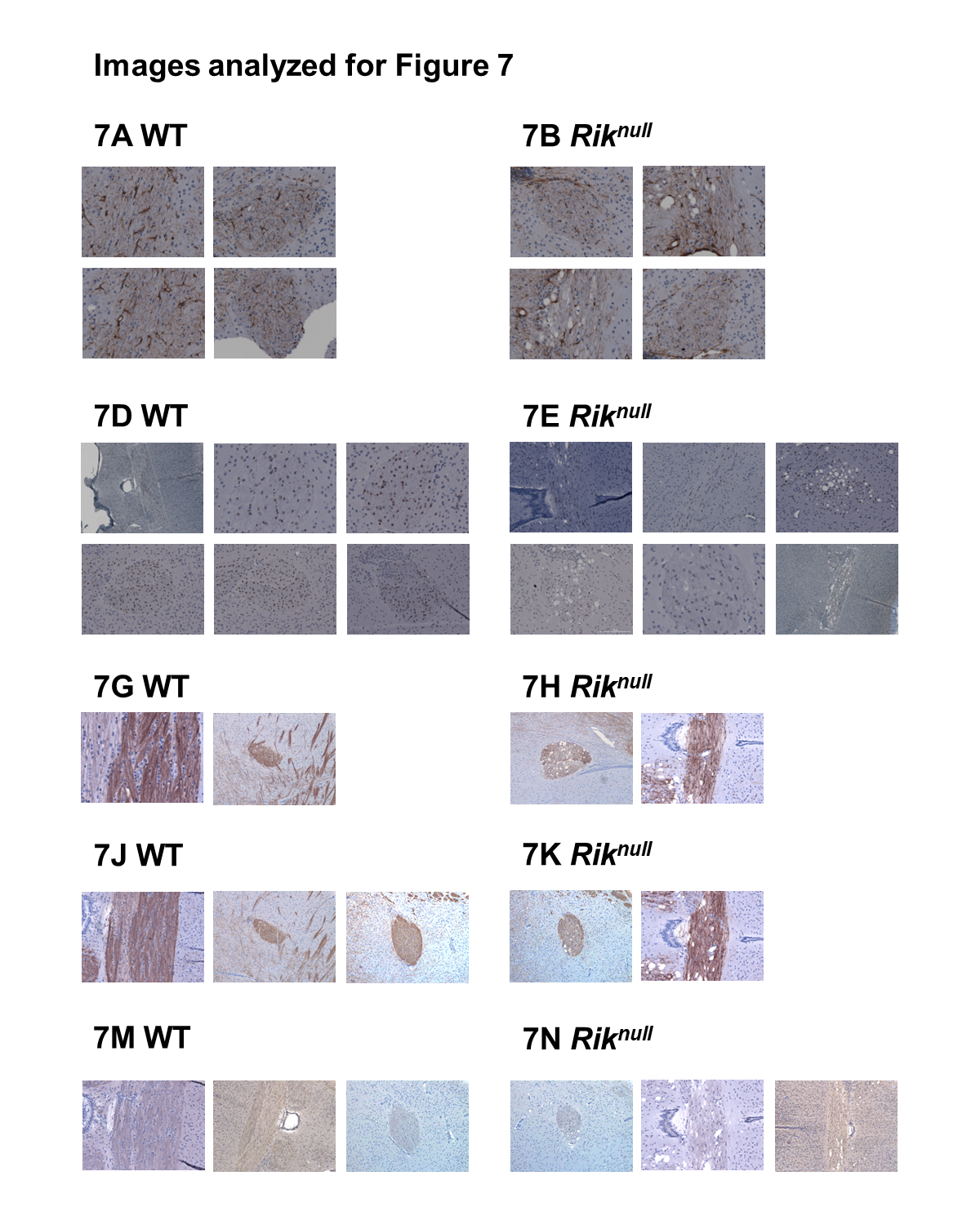

Supplement: S10 Fig — (TIF) [file pone.0290487.s010.tif]
